# The influence of rivers on seabird foraging ecology

**DOI:** 10.1002/brv.70143

**Published:** 2026-02-10

**Authors:** Julia B. Morais, Andre Chiaradia, Richard D. Reina

**Affiliations:** ^1^ School of Biological Sciences, Monash University Wellington Rd Clayton VIC 3800 Australia; ^2^ Conservation Department, Phillip Island Nature Parks 154/156 Thompson Ave Cowes VIC 3922 Australia

**Keywords:** seabirds, river plumes, estuaries, hydrological influences, marine food webs, prey accessibility, flow, turbidity, climate change, resource buffer

## Abstract

Rivers act as vital arteries to the world's oceans, delivering fresh water and nutrients that sustain marine ecosystems. Globally, river flow increasingly is being altered by climate change and anthropogenic pressures; yet the significance of rivers to predatory marine species, such as seabirds, and the extent to which river‐related changes affect their food webs, remains poorly understood. This review synthesises 51 studies specifically designed to examine river influences on seabird habitat selection, diet, health, and demographics, while highlighting methodological approaches and ecological patterns. Although river‐related variables remain underutilised in seabird research, 88% (45/51) of studies that included them reported clear evidence of river effects for at least one type of seabird response, suggesting these ecological links are under‐recognised rather than absent. When selected as the primary explanatory (most informative) variable in each study, plume‐based metrics were conclusive in 95% (19/20) of cases, whereas river‐specific metrics (namely number of rivers, river outflow and distance to river mouths) were conclusive in 84% (26/31) of cases, confirming that both metric types are highly reliable once aligned with the seabird response in question. River‐influenced coastal waters consistently supported critical foraging hotspots across all seabird orders, whilst also exposing birds to potential pollutant burdens and altered prey dynamics. Seabird dietary data are a valuable indicator of prey variability associated with river outflows, with greater prey diversity recorded in estuarine habitats compared to marine ones. Rivers exhibited mixed effects under anthropogenic pressures but generally positive influences during climate disturbances, suggesting that seabirds may increasingly depend on riverine environments as buffers against changing marine conditions. We recommend expanding investigations into river impacts on seabird health in tropical systems, incorporating long‐term hydrological influences, and prioritising the integration of river‐specific and oceanographic data to predict seabird responses more effectively in a rapidly changing world.

## I. INTRODUCTION

As the main tributaries of freshwater and terrestrial nutrients into the sea, rivers support marine food webs and keep marine ecosystems resilient (O'Leary *et al*., 2017; Hansen *et al*., 2023). In healthy coastal environments, freshwater outflows promote ocean productivity and form estuarine and river plume fronts, where plankton aggregates in nutrient‐rich and light‐exposed waters (Gillanders & Kingsford, 2002; Naselli‐Flores & Padisák, 2023). Enhanced bioproductivity attracts large numbers of zooplankton and forage fish to these areas, frequently constituting seascape nurseries (Nagelkerken *et al*., 2015; Fan *et al*., 2023). In many Large Marine Ecosystems (LMEs) (Sherman & Hamukuaya, 2016), river plumes and estuaries are important permanent or alternative foraging hotspots for piscivorous predators, often coinciding with fisheries grounds (Belkin & Cornillon, 2007; Broadley *et al*., 2022). However, emerging evidence suggests that the influence of riverine inputs on the world's oceans has likely been underestimated (Auricht *et al*., 2022; Broadley *et al*., 2022). As such, coastal ecosystems may disproportionately bear the consequences of anthropogenic activities impacting rivers, with implications across all trophic levels and ecosystem services (Maavara *et al*., 2020; Malone & Newton, 2020).

Changes in freshwater runoff alter nutrient availability, sediment loads, pH, temperature, salinity, water clarity, and circulation patterns, all of which influence ocean foraging conditions for marine predators in these coastal food webs (Fasola *et al*., 1989; Cloern *et al*., 2017; Beavis *et al*., 2023; Gupta, Reddy & Gandla, 2023). While river discharge naturally fluctuates due to seasonal and geographical factors, creating intrinsic variability in ocean conditions to which predators are adapted, extrinsic variability from extreme weather disturbances such as droughts, storms, and floods is becoming more frequent and severe with climate change. When combined with other human‐driven changes to river systems such as hydropower, storage dams, land use, and other managed alterations, the combined pressures can disrupt sediment transport, nutrient cycling, salinity balance, and other conditions upon which coastal marine ecosystems depend (Dias *et al*., 2019b; Orgeret *et al*., 2022), potentially pushing these systems beyond their adaptive capacity to withstand disturbance (Foden *et al*., 2019).

Pollution exacerbates these challenges, as rivers transport contaminants from industrial, agricultural, and mining sources to the ocean. Natural sediment transport plays a key role in sustaining coastal habitats, but excessive, human‐driven inputs degrade ecosystems and lead to the accumulation of pollutants in marine food webs (Ryan, 2015; Atwood *et al*., 2019; Van Sebille *et al*., 2020; Ponton *et al*., 2021), with persistent and pervasive effects rather than direct changes to river systems. Extrinsic factors such as heavy storms and large‐scale anthropogenic actions may exacerbate marine pollution, leading to concentrated pollutant loads in estuaries (Drinkwater & Frank, 1994; Provencher *et al*., 2017) and the mass release of contaminants into seabird habitats (Nunes *et al*., 2022; de Barros Bauer *et al*., 2024). As climate change progressively alters the hydrological cycle, and human population growth increases pollution rates and demand for water and related resources, monitoring the effects of river‐to‐sea interactions on downstream marine predators becomes critical (Couto & Sethi, 2024).

Seabirds are mid to top predators regarded as indicators of marine food supplies (Piatt *et al*., 2007) and sentinels of coastal ecosystems (Boersma, 2008; Wolf *et al*., 2010; Rodríguez *et al*., 2019; Ropert‐Coudert *et al*., 2019; Lieber, Langrock & Nimmo‐Smith, 2021). Moreover, this group is considered the easiest to study among marine animals due to their visibility at sea and dependence on land for breeding (Assali, Bez & Tremblay, 2020; Power *et al*., 2023). Our understanding of seabird foraging ecology is largely based on data collected during the breeding season, when birds remain closer to their colonies on land and are more likely to interact with terrestrial features such as estuaries and river plumes (Fasola *et al*., 1989; Shealer, 2002). These coastal features can shape foraging decisions by altering prey availability and/or accessibility (Skov & Prins, 2001; Markones, 2007; Royan *et al*., 2014; Cox *et al*., 2018), and sustained benefits may even influence breeding site selection over time. Emerging evidence of diet shifts and localised recruitment near rivers – observed in both philopatric and non‐philopatric species – suggests a potential for growing ecological dependencies on river‐influenced systems under changing marine conditions (Jodice & Suryan, 2010; Kowalczyk, 2015; Coulson, 2016; Poupart *et al*., 2017; Brisson‐Curadeau & Elliott, 2019; Price *et al*., 2020; Evans, Lea & Hindell, 2021; Soanes *et al*., 2021; Lerma *et al*., 2022; Seher *et al*., 2022).

Although the importance of rivers has been established for inland‐breeding seabirds and waterbirds, and the collection of river flow data is increasingly encouraged in climate change impact assessments (Royan *et al*., 2014; Royan, 2015; O'Keeffe *et al*., 2023), the interplay between seabirds and coastal river systems has often been overlooked. This may stem from the historical separation of seabird studies from terrestrial sciences into the domain of marine science, which has led to rivers being viewed primarily as inland features influencing breeding ecology, rather than as drivers of coastal ecosystem productivity affecting seabird foraging ecology (Ainley, Ribic & Woehler, 2012). Unlike terrestrial ecosystems, the marine environment consists of highly dynamic habitats with shifting boundaries and short‐lived structures, which are expected to become increasingly unpredictable in the future (Carr *et al*., 2003; Weimerskirch, 2007; Ropert‐Coudert, Kato & Chiaradia, 2009; Burrows *et al*., 2011; Webb, 2012; d'Alcalà, 2019). Conversely, regions of freshwater influence and associated river plume fronts may persist throughout the year, varying primarily in location or extent. These areas may be targeted year round by seabirds, offering more reliable foraging opportunities than many marine habitats (Skov & Prins, 2001; Scales *et al*., 2014; Broadley *et al*., 2022; Kralj *et al*., 2025).

While a fairly large body of literature addresses the impact of river plume dynamics on marine organisms in lower trophic levels (Gillanders & Kingsford, 2002; Broadley *et al*., 2022), a comprehensive review detailing the impacts on predatory species, such as seabirds, is lacking. This review aims to fill that gap by synthesising existing studies that examine how rivers influence seabird foraging ecology. We categorise responses into four main types (habitat selection, diet, health, and demographics) and assess the prevalence and significance (i.e. conclusive evidence) of river effects. In doing so, we aim to reposition rivers as ecologically significant coastal features in seabird ecology and conservation. We also investigate the factors most associated with detecting and reporting the effects of rivers on seabirds, such as taxon, region, study duration, selected environmental variables and river metrics. Finally, we consider how river–seabird relationships may shift under extrinsic disturbances such as marine heatwaves, extreme weather and pollution events, particularly in light of climate change and intensifying human impacts on watersheds.

## II. METHODS

### (1) Literature review protocol and scope

**Fig. 1:**
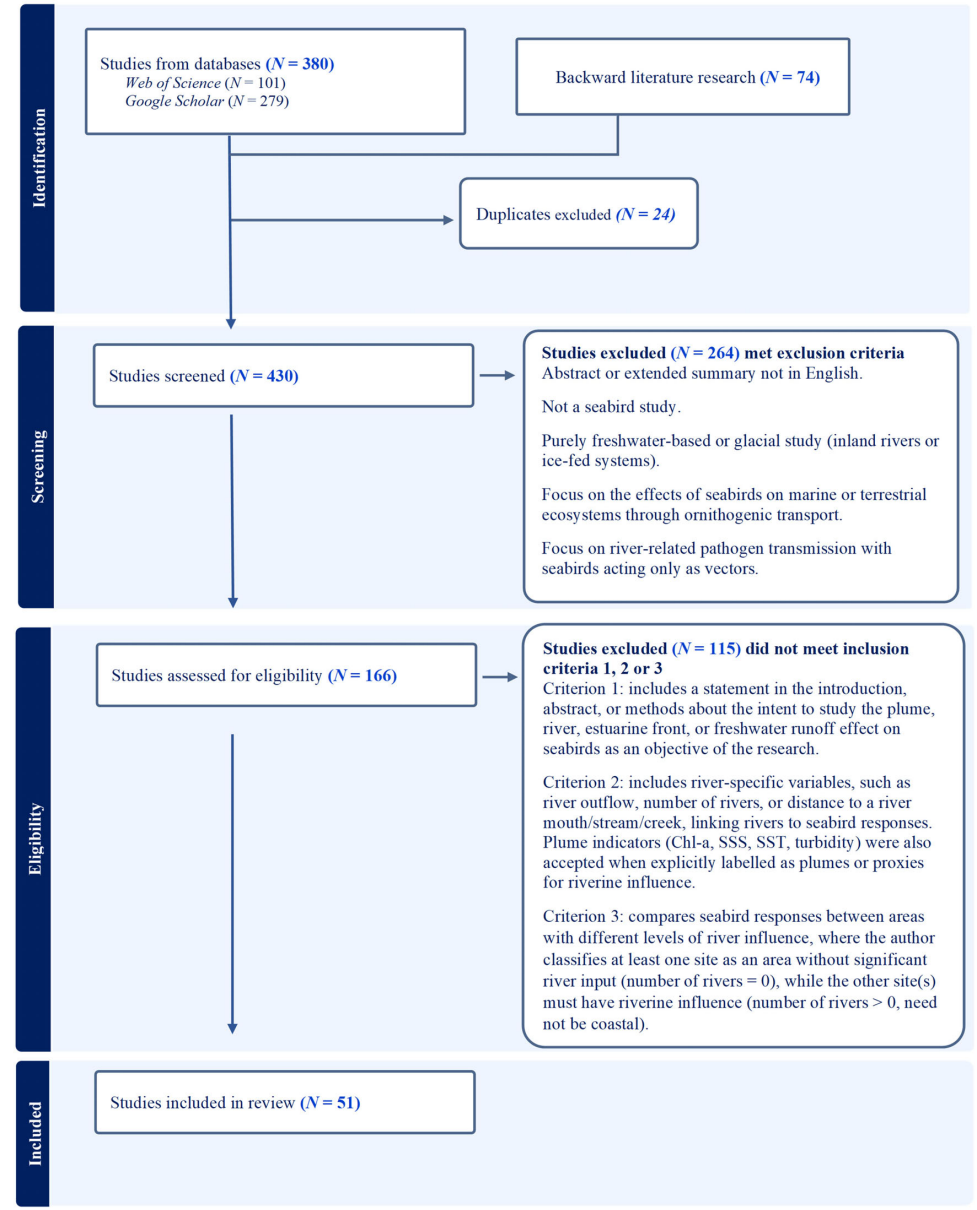
PRISMA flow chart detailing the process of record collection and study selection. See Table S1 for information used to include/exclude each study from the review. Chl‐a, chlorophyll‐a concentration; SSS, sea surface salinity; SST, sea surface temperature.

We conducted a systematic literature search following the PRISMA 2020 guidelines (Page *et al*., 2021) to identify studies examining the effects of rivers on seabird foraging ecology. The PRISMA flow diagram is presented in Fig. 1, and the full list of search terms used is provided in Table 1.

**Table 1:** Full list of search terms used in *Web of Science* and *Google Scholar* databases. *Google Scholar* searches were performed using *Publish or Perish* software with a maximum result limit of 1000.

Database	Target population		Freshwater source		Interaction outcomes		Other freshwater sources	Refined by
*Web of Science*	‘seabird*’	**AND**	‘river’	**AND**	‘forag*’	**NOT**	‘fjord’	‘seabird*’
	‘marine bird*’		‘plume’		‘diet*’		‘ice’	‘forag*’
	‘avian’		‘estuar*’		‘behav*’			‘river’
	‘marine vertebrate*’		‘watershed’		‘distrib*’			
	‘marine top‐predator*’		‘stream*’		‘breed*’			
	‘ocean* top predator*’		‘creek*’		‘respon*’			
			‘*flow*’		‘surviv*’			
			‘freshwater’		‘demograph*’			
			‘fluvial*’		‘phenolog*’			
			‘discharge*’		‘dynamic*’			
			‘runoff’		‘health’			
*Google Scholar*	‘seabird’		‘river’		‘forage’		‘fjord’	
			‘plume’				‘ice’	
			‘estuary’					

By 12 March 2025, the search retrieved 380 relevant records. An additional 74 studies were identified through backward citation searching (Harari *et al*., 2020), bringing the total to 430 publications after exclusion of duplicates. Each study was assessed using predefined criteria [Fig. 1; *N* = 166 studies after initial screening (see online Supporting Information, Table S1); *N* = 51 studies after applying detailed screening criteria (see Table S2 for list of included studies)] to select those that, based on their design and objectives, investigated the effects of river systems on seabird foraging ecology. Studies that did not meet the inclusion criteria were considered to offer only circumstantial evidence and were excluded, along with those presenting results in unusable formats (e.g. data sets). Records without an English abstract or extended summary were also excluded, as core findings could not be reliably assessed.

**Fig. 2:**
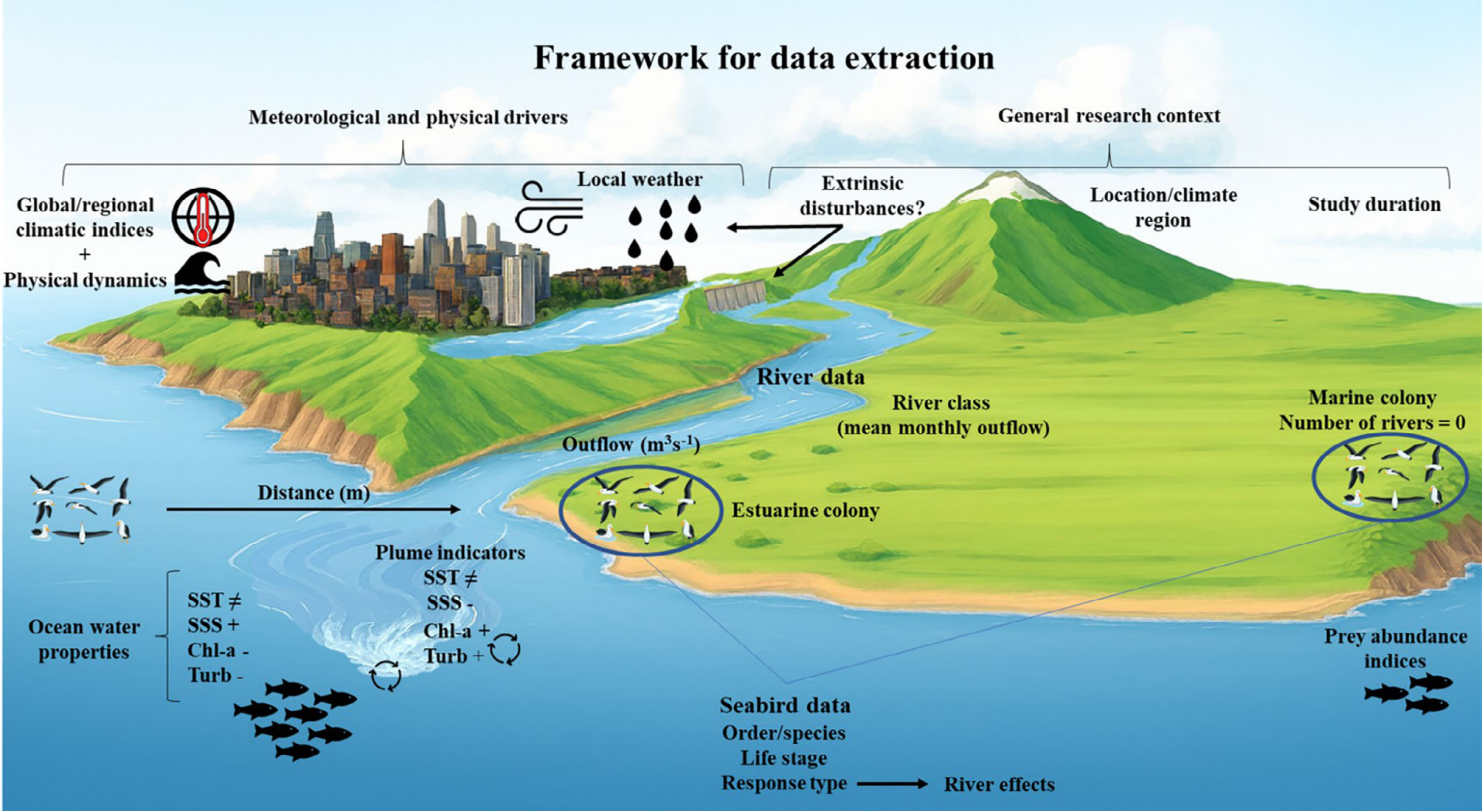
Conceptual framework for data extraction, illustrating the rationale and scope of information systematically collected from each study to assess the influence of rivers on seabird ecology. Chl‐a, chlorophyll‐a concentration; River class according to Meijer *et al*. (2021); SSS, sea surface salinity; SST, sea surface temperature; Turb, turbidity.

As part of defining the review scope, we developed a conceptual framework to identify and integrate key ecological components of the river–seabird relationship, while guiding the systematic extraction of information from each study (Fig. 2).

### (2) General research characteristics

From each selected study, we extracted general study characteristics informing about the research context, including geographic region, climate regime, study duration, river name, and river size class (following Meijer *et al*., 2021). River size was categorised based on mean monthly discharge: very small (<10 m^3^ s^−1^), small (10–100 m^3^ s^−1^), medium (100–1,000 m^3^ s^−1^), large (1,000–10,000 m^3^ s^−1^) and very large (>10,000 m^3^ s^−1^). Study duration was recorded in years and classified as short term (≤4 years), medium term (5–19 years), or long term (≥20 years) according to Hughes *et al*. (2017) and Lindenmayer *et al*. (2012).

Geographical locations were assigned to LMEs, and climate regions were used to infer river flow regimes (e.g. temperature *versus* rainfall dominated) based on Zeiringer *et al*. (2018). We also recorded seabird taxonomy, breeding status, and International Union for Conservation of Nature (IUCN) population status (IUCN, 2025), with *Red List* categories assigned at the species level and matched across sources by species epithet, thereby enabling assessments of foraging strategies, life‐stage‐specific responses, and knowledge gaps. Extrinsic disturbances such as storms, droughts, or pollution events were noted according to the IUCN threats classification scheme (Dias *et al*., 2019b, IUCN, 2025). Where such events were not reported, we assumed that studies were conducted under intrinsic variability. Pollution was only considered an extrinsic disturbance when clearly exacerbated by external factors (e.g. dam collapse).

These contextual variables provided ecological background but were not used as explanatory variables for river influence.

### (3) Environmental variables

Environmental variables were extracted for their potential explanatory or modulating value and included meteorological and physical drivers, prey‐related variables, and river metrics, expressed either as river‐specific variables or as plume indicators derived from ocean water properties.

Meteorological and physical drivers included global and regional climate indices (e.g. Southern Annular Mode, Southern Oscillation Index), local weather variables (e.g. wind, rainfall), and physical oceanographic processes (e.g. tides, upwelling, currents). These multi‐scale forcing mechanisms shape ocean structure, productivity, and prey availability, and were considered as modulators of seabird responses.

Prey variables captured direct indicators of food availability, through fish‐based prey abundance indices, providing concurrent measures of the prey stock available to seabirds during the study period. Where prey data were available, we also recorded the methods used, in order to assess how prey metrics were incorporated in the interpretation of river effects. Although these modulating variables influence the overall environmental conditions that seabirds experience, they were not considered primary evidence of river influence. Environmental variables were extracted only when explicitly reported in the analysis or when used quantitatively to interpret seabird responses, regardless of whether a formal statistical test was applied.

### (4) River metrics and evidence type

Variables indicating the presence and influence of rivers (river metrics) were classified as either river‐specific or non‐river‐specific, depending on their ability to attribute observed seabird responses to freshwater discharge.

For river‐specific variables, we included: (*i*) *River outflow/discharge* (m^3^ s^−1^) – direct measurements of the volume of freshwater entering the coastal system from rivers. We used the terms ‘outflow’ and ‘discharge’ interchangeably to describe water flow over time, acknowledging that terminology may vary across disciplines; (*ii*) *number of rivers* – spatial count of freshwater sources within the study area, this variable could be used as ordinal, weighted, or together with a control (binary); and (*iii*) *distance to river* – linear distances between seabird observation points (e.g. colonies or at‐sea locations) and to variable‐size river outlets (river mouths, estuaries, creeks, streams).

For non‐river‐specific variables we recorded several *plume indicators*. River plumes are buoyant surface‐layer features formed by the discharge of fresh water, sediments, nutrients, and organic matter into coastal zones. These inputs create distinct hydrographic structures characterised by altered ocean water properties: reduced sea surface salinity (SSS), elevated turbidity, higher chlorophyll‐a concentration (Chl‐a), and sometimes distinct sea surface temperature (SST) patterns compared to surrounding marine waters (Fig. 2). Although these ocean‐water properties are frequently used to indicate or describe river plume influence and can be embedded into plume metrics or indices (e.g. salinity or temperature thresholds used to delineate plume area), they were classified as non‐river‐specific because similar patterns can arise from other oceanographic processes, including tidal mixing, wind‐driven upwelling, and mesoscale eddies, making these variables non‐exclusive indicators of freshwater input. At the boundaries of river plumes, known as plume fronts, mixing between fresh water and sea water can lead to localised prey aggregation, attracting foraging seabirds (Gillanders & Kingsford, 2002).

Static bathymetric variables were excluded from analysis, as the review focused on dynamic water properties. For the purposes of this review, we used the terms ‘turbidity’ and ‘water clarity’ interchangeably, despite methodological differences in their measurement.

Further, based on the use of the above‐mentioned river metrics, each study was assessed to determine the strength of evidence it provided for river influence. The following evidence types were considered: conclusive, not significant, or inconclusive. Details of the research findings that supported classification for each study are provided in Table S3.

Unless stated otherwise, river metrics were considered as evidence for river effects when supported by clear author interpretation, statistically significant results or both (Table 2). Both river‐specific and non‐river‐specific variables were classified as *inconclusive* when the evidence for river effects was unclear, not directly tested, or not explicitly interpreted by the study authors. This included studies in which river‐related variables were incorporated into multivariate models (e.g. principal components analysis, PCA) but not examined individually, or when variation in the river metric occurred only temporally without direct analysis (e.g. simple comparisons of seabird responses between years with differing river conditions). In such cases, conclusions about river influence could not be robustly inferred.

**Table 2:** Summary of studies designed to investigate river effects on seabird responses across the different taxa and the river metrics used to research this relationship.

River effects	Common name	Life stage^1^	River name and class^2^	River metric^3^	Study duration^4^ (years)	Extrinsic disturbances^5^ (Y/N)	Large Marine Ecosystem^6^	Reference
CHARADRIIFORMES								
**Habitat** **selection**	Arctic tern, common tern	Br	Elbe (M)	Distance (not significant)	5	N	North Sea	Schwemmer *et al*. (2009)
	Common murre, razorbills, unidentified alcids	NBr	Vilaine, Adour, Loire, Gironde (S‐L)	Plume/Chl‐a, SSS, SST	10	N	Celtic‐Biscay Shelf	Lambert *et al*. (2018)
	Black tern, Bonaparte's gull, bridled tern, brown noddy, common tern, herring gull, laughing gull, least tern, royal tern, Sandwich tern, sooty tern	NS	Mississippi (VL)	Plume/Chl‐a, SSS, SST	2	Y (flood)	Gulf of Mexico	Ribic *et al*. (1997)
	Arctic tern, black‐legged kittiwake, common gull, common tern, Sandwich tern	Br	Elbe (M)	Plume/SST, SSS, Turb	4	N	North Sea	Markones (2007)
	Common murre, herring gull, lesser black‐backed gull	Br	Elbe (M)	Plume/SST, SSS, Turb (not significant)	4	N	North Sea	Markones (2007)
	Great black‐backed‐gull, razorbill	Br	Elbe (M)	Plume/SST, SSS, Turb (inconclusive)	4	N	North Sea	Markones (2007)
	Caspian tern	Br	Columbia (L)	Plume/SSS‐salinity zones	2	Y (colony relocation)	California Current	Anderson *et al*. (2007)
	Common murre	Br	Columbia (L)	Plume/SSS, SST	4	N	California Current	Zamon *et al*. (2014)
	Common murre	Br	Columbia (L)	Plume/SSS, SST, Turb	3	N	California Current	Phillips *et al*. (2017)
	Common murre	Br	Columbia (L)	Plume/SSS	2	N	California Current	Phillips *et al*. (2018)
	Common murre	NBr	Columbia (L)	Plume/Chl‐a, SSS‐salinity class, SST	3	Y (marine heatwave)	California Current	Loredo *et al*. (2019)
	Common murre	NS	Columbia (L)	Plume/SSS, Turb	3	N	California Current	Phillips *et al*. (2021)
	Marbled murrelet	Br	Creeks (VS)	Distance	68; 22	N	Gulf of Alaska	Yen *et al*. (2004)
	Marbled murrelet	Br	Creeks, streams (VS)	Number/weighted	3	N	Gulf of Alaska	Barrett (2008)
	Marbled murrelet	Br	Creeks, Speel, Whiting (VS‐S)	Distance	1	N	Gulf of Alaska	Haynes *et al*. (2011)
	Marbled murrelet	Br	Creeks, Klamath, Smith, San Joaquin, Columbia (VS‐L)	Distance	13	N	California Current	Raphael *et al*. (2015)
	Marbled murrelet	Br	Creeks (VS)	Distance	2	N	Gulf of Alaska	Pastran *et al*. (2021)
	Marbled murrelet	Br	Creeks, Yaquina, Nestucca, Siuslaw (VS–S)	Number/weighted	3	N	California Current	Garcia‐Heras *et al*. (2024)
	Pomarine skua	NS	Mississippi (VL)	Plume/Chl‐a, SSS, SST (not significant)	2	Y (flood)	Gulf of Mexico	Ribic *et al*. (1997)
	Slender‐billed gull	NS	Ebro (M)	Plume/Chl‐a, SST	2	N	Mediterranean Sea	Cama *et al*. (2012)
	Yellow‐legged gull	Br	Minho (M)	Plume/SSS	1	Y (storm, flood)	Iberian Coastal	Waggitt *et al*. (2020)
**Diet/feeding behaviour**	Artic tern, common tern	Br	Elbe (M)	Distance (not significant)	5	N	North Sea	Schwemmer *et al*. (2009)
	Caspian tern	Br	Columbia (L)	Outflow (inconclusive)	2	Y (colony relocation)	California Current	Anderson *et al*. (2007)
	Caspian tern	Br	Columbia (L)	Outflow	11	N	California Current	Collar *et al*. (2017)
	Caspian tern	Br	Columbia (L)	Outflow	10	Y (colony relocation)	California Current	Lyons (2010)
	Common murre, glaucous gull, glaucous‐winged gull, thick‐billed murre	Br (egg)	Yukon (L)	Number/control	7	N	East Bering Sea & Gulf of Alaska	Day *et al*. (2012)
	Common murre	Br	Yaquina (S)	Number/control	10	N	California Current	Gladics *et al*. (2015)
	Common murre	NBr	Columbia (L)	Plume/Chl‐a, SSS‐salinity class, SST	3	Y (marine heatwave)	California Current	Loredo *et al*. (2019)
	Common tern	NBr	La Plata (VL)	Outflow	7	N	Patagonian Shelf	Mauco & Favero (2005)
	Common tern	Br	Drava, Sava (M)	Number/control	2	N	Mediterranean Sea	Kralj *et al*. (2025)
	Fairy tern	Br	Swan‐Canning (M)	Number/control	3	N	West Central Australian Shelf	Greenwell *et al*. (2021)
	Great black‐backed gull, herring gull, lesser black‐backed gull	Br	Seine (M)	Number/control	3	N	Celtic‐Biscay Shelf	Binkowski *et al*. (2021)
	Herring gull, laughing gull, royal tern, Sandwich tern	NBr (deceased juvenile)	Cape Fear, >1 BSUR (VS‐M)	Number/control	1	N	Northeast U.S. Continental Shelf	Robuck *et al*. (2020)
	Pigeon guillemot	Br	Petaluma, Napa, Guadalupe, San Joaquin, Sacramento (VS‐M)	Number/control	3	Y (marine heatwave)	California Current	Seher *et al*. (2022)
**Health and disease**	Ancient murrelet, glaucous‐winged gull, rhinoceros auklet	Br (egg)	Fraser (L)	Number/control (inconclusive)	16	N	Gulf of Alaska	Elliott *et al*. (1989)
	Blackheaded gull, yellow‐legged gull	NS	Alvares/Avilés, Linares/Villaviciosa, Eo (VS‐M)	Number/control (inconclusive)	1	N	Celtic‐Biscay Shelf	Masiá *et al*. (2019)
	Common murre, glaucous gull, glaucous‐winged gull, thick‐billed murre	Br (egg)	Yukon (L)	Number/control	7	N	East Bering Sea & Gulf of Alaska	Day *et al*. (2012)
	Great black‐backed gull, herring gull, lesser black‐backed gull	Br	Seine (M)	Number/control (inconclusive)	3	N	Celtic‐Biscay Shelf	Binkowski *et al*. (2021)
	Herring gull, laughing gull, royal tern, Sandwich tern	NBr (deceased juvenile)	Cape Fear, >1 BSUR (VS‐M)	Number/control	1	N	Northeast U.S. Continental Shelf	Robuck *et al*. (2020)
**Demographics**	Black‐legged kittiwake, common murre	Br	River Forth (S)	Outflow (inconclusive)	20–30	Y (industrial fishery)	North Sea	Scott *et al*. (2006)
	Cabot's tern, kelp gull	NS (deceased)	São Jorge, Macaé, Paraíba, Doce (S‐L)	Outflow	4	Y (storm)	East Brazil Shelf	Tavares *et al*. (2016)
	Caspian tern	Br	Columbia (L)	Outflow (inconclusive)	2	Y (colony relocation)	California Current	Anderson *et al*. (2007)
	Caspian tern	Br	Columbia (L)	Outflow (not significant)	10	Y (colony relocation)	California Current	Lyons (2010)
	Caspian tern	Br	Columbia (L)	Outflow	11	N	California Current	Collar *et al*. (2017)
	Common murre	Br	Yaquina (S)	Number/control	10	N	California Current	Gladics *et al*. (2015)
	Pigeon guillemot	Br	Petaluma, Napa, Guadalupe, San Joaquin, Sacramento (VS‐M)	Number/control	3	Y (marine heatwave)	California Current	Seher *et al*. (2022)
GAVIIFORMES						
**Habitat selection**	Black‐throated diver, red‐throated diver	NBr	Elbe (M)	Plume/Chl‐a, SSS, SST, Turb	8	N	North Sea	Skov & Prins (2001)
PELICANIFORMES						
**Habitat selection**	Brown pelican	Br & NBr	Rio Grande, Colorado, Guadalupe, Brazos, Suwannee, Mississippi (S‐VL)	Plume/SSS, SST	3	N	Gulf of Mexico	Lamb *et al*. (2020)
**Diet/feeding behaviour**	Brown pelican	NBr (deceased juvenile)	Cape Fear, >1 BSUR (VS‐M)	Number/control	1	N	Northeast U.S. Continental Shelf	Robuck *et al*. (2020)
**Health and disease**	Brown pelican	NBr (deceased juvenile)	Cape Fear, >1 BSUR (VS‐M)	Number/control	1	N	Northeast U.S. Continental Shelf	Robuck *et al*. (2020)
PHAETHONTIFORMES						
**Habitat selection**	Red‐billed tropicbird	NS	Doce (L)	Plume/SST (inconclusive)	4	Y (dam collapse)	East Brazil Shelf	Nunes *et al*. (2022)
**Diet/feeding behaviour**	Red‐billed tropicbird	NS	Doce (L)	Plume/SST (inconclusive)	4	Y (dam collapse)	East Brazil Shelf	Nunes *et al*. (2022)
**Health and disease**	Red‐billed tropicbird	NS	Doce (L)	Plume/SST (inconclusive)	4	Y (dam collapse)	East Brazil Shelf	Nunes *et al*. (2022)
PROCELLARIIFORMES						
**Habitat selection**	Audubon's shearwater, band‐rumped storm‐petrel, Cory's shearwater	NS	Mississipi (VL)	Plume/SSS, Chl‐a, SST	2	Y (flood)	Gulf of Mexico	Ribic *et al*. (1997)
	Balearic shearwater	NBr	Guadiana, Guadalquivir (M)	Distance	10	N	Iberian Coastal	de la Cruz *et al*. (2021)
	Barau's petrel	Br	336 rivers (VS‐M)	Outflow (inconclusive)	2	N	Agulhas Current	Thibault *et al*. (2024)
	Northern fulmar	Br	Elbe (M)	Plume/SST, SSS, Turb (not significant)	4	N	North Sea	Markones (2007)
	Northern fulmar	NBr	Vilaine, Adour, Loire, Gironde (S‐L)	Plume/Chl‐a, SSS, SST	10	N	Celtic‐Biscay Shelf	Lambert *et al*. (2018)
	Sooty shearwater	NBr	Columbia (L)	Plume/SSS, SST	4	N	California Current	Zamon *et al*. (2014)
	Sooty shearwater	NBr	Columbia (L)	Plume/SSS, SST, Turb	3	N	California Current	Phillips *et al*. (2017)
	Sooty shearwater	NBr	Columbia (L)	Plume/SSS	2	N	California Current	Phillips *et al*. (2018)
	Sooty shearwater	NS	Columbia (L)	Plume/SSS, Turb	3	N	California Current	Phillips *et al*. (2021)
	Streaked shearwater	Br	Changjiang (VL)	Plume/Chl‐a, SST	1	N	East China Sea	Matsumoto *et al*. (2016)
	Trindade petrel	NS	Doce (L)	Plume/SST (inconclusive)	4	Y (dam collapse)	East Brazil Shelf	Nunes *et al*. (2022)
	Wedge‐tailed shearwater	Br	Calliope (S)	Plume/Chl‐a, SST, Turb	2	N	Northeast Australian Shelf	McDuie *et al*. (2018)
**Diet/feeding behaviour**	Great shearwater	NBr (deceased juvenile)	Cape Fear, >1 BSUR (VS‐M)	Number/control	1	N	Northeast U.S. Continental Shelf	Robuck *et al*. (2020)
	Trindade petrel	NS	Doce (L)	Plume/SST (inconclusive)	4	Y (dam collapse)	East Brazil Shelf	Nunes *et al*. (2022)
**Health and disease**	Barau's petrel	Br	336 rivers (VS‐M)	Outflow (inconclusive)	2	N	Agulhas Current	Thibault *et al*. (2024)
	Fork‐tailed storm‐petrel, Leach's storm‐petrel	Br (egg)	Fraser (L)	Number/control (inconclusive)	16	N	Gulf of Alaska	Elliott *et al*. (1989)
	Great shearwater	NBr (deceased juvenile)	Cape Fear, >1 BSUR (VS‐M)	Number/control	1	N	Northeast U.S. Continental Shelf	Robuck *et al*. (2020)
	Trindade petrel	NS	Doce (L)	Plume/SST (inconclusive)	4	Y (dam collapse)	East Brazil Shelf	Nunes *et al*. (2022)
**Demographics**	Audubon's shearwater	Br & NBr	Orinoco, Amazon (VL)	Outflow	20	N	North Brazil Shelf	Precheur *et al*. (2016)
SPHENISCIFORMES						
**Habitat selection**	Little penguin	Br	Yarra (S)	Plume/Chl‐a, SSS, SST	3	Y (drought)	Southeast Australian Shelf	Kowalczyk *et al*. (2015a)
	Little penguin	Br	Yarra (S)	Plume/Chl‐a, SSS, SST, Turb	3	N	Southeast Australian Shelf	Kowalczyk *et al*. (2015b)
	Little penguin	Br	Nile, Hutt, Buller (S‐M)	Distance	6	N	New Zealand Shelf	Poupart *et al*. (2017)
**Diet/feeding behaviour**	Little penguin	Br	Yarra (S)	Plume/Chl‐a, SSS, SST	3	Y (drought)	Southeast Australian Shelf	Kowalczyk *et al*. (2015a)
	Little penguin	Br	Nile, Hutt, Buller (S‐M)	Distance	6	N	New Zealand Shelf	Poupart *et al*. (2017)
**Health and disease**	Magellanic penguin	Br	La Plata (VL)	Plume/SST	31	N	Patagonian Shelf	Rebstock & Boersma (2018)
**Demographics**	Little penguin	Br	Yarra (S)	Plume/Chl‐a, SSS, SST	3	Y (drought)	Southeast Australian Shelf	Kowalczyk *et al*. (2015a)
	Little penguin	Br	Nile, Hutt, Buller (S‐M)	Distance (inconclusive)	6	N	New Zealand Shelf	Poupart *et al*. (2017)
	Little penguin	Br & NBr	Murray (M)	Outflow	38; 16	Y (drought)	Southeast Australian Shelf	Colombelli‐Négrel *et al*. (2022)
	Magellanic penguin	Br	La Plata (VL)	Plume/SST	31	N	Patagonian Shelf	Rebstock & Boersma (2018)
	Magellanic penguin	Br & NBr	La Plata (VL)	Plume/SST	38	Y (marine heatwave, storms, floods)	Patagonian Shelf	Clark‐Wolf *et al*. (2023)
SULIFORMES						
**Habitat selection**	Brown booby	NS	Doce (L)	Plume/SST (inconclusive)	4	Y (dam collapse)	East Brazil Shelf	Nunes *et al*. (2022)
	European shag	Br	Minho (M)	Plume/SSS	1	Y (storm, flood)	Iberian Coastal	Waggitt *et al*. (2020)
	Magnificent frigatebird, masked booby, northern gannet	NS	Mississippi (VL)	Plume/Chl‐a, SSS, SST	2	Y (flood)	Gulf of Mexico	Ribic *et al*. (1997)
	Northern gannet	Br	Elbe (M)	Plume/SST, SSS, Turb (not significant)	4	N	North Sea	Markones (2007)
	Northern gannet	NBr	Vilaine, Adour, Loire, Gironde (S‐L)	Plume/Chl‐a, SSS, SST	10	N	Celtic‐Biscay Shelf	Lambert *et al*. (2018)
**Diet/feeding behaviour**	Brown booby	NS	Doce (L)	Plume/SST (inconclusive)	4	Y (dam collapse)	East Brazil Shelf	Nunes *et al*. (2022)
	Double‐crested cormorant	Br	Columbia (L)	Outflow	10	Y (colony relocation)	California Current	Lyons (2010)
	Double‐crested cormorant	Br	Columbia (L)	Outflow	15	N	California Current	Lyons *et al*. (2014)
	Great cormorant	NS	Dee, Wye, Severn (S)	Number/control	3	N	Celtic‐Biscay Shelf	Bearhop *et al*. (1999)
	Great cormorant	NS	Minho (M)	Outflow	3	N	Iberian Coastal	Dias *et al*. (2012)
	Great cormorant	NBr	Segura (S)	Number/control	2	N	Mediterranean Sea	Farinós‐Celdrán *et al*. (2019)
**Health and disease**	Brown booby	NS	Doce (L)	Plume/SST (inconclusive)	4	Y (dam collapse)	East Brazil Shelf	Nunes *et al*. (2022)
	Double crested cormorant, pelagic cormorant	Br (egg)	Fraser (L)	Number/control (inconclusive)	16	N	Gulf of Alaska	Elliott *et al*. (1989)
	European shag	NS	Alvares/Avilés, Linares/Villaviciosa, Eo (VS‐M)	Number/control (inconclusive)	1	N	Celtic‐Biscay Shelf	Masiá *et al*. (2019)
	Guanay cormorant	NS	SUR (VS‐S)	Number/ordinal	2	N	Humboldt Current	Diaz‐Santibañez *et al*. (2023)
**Demographics**	Brown booby, magnificent frigatebird	NS (deceased)	São Jorge, Macaé, Paraíba, Doce (S‐L)	Outflow	4	Y (storm)	East Brazil Shelf	Tavares *et al*. (2016)
	Double‐crested cormorant	Br	Columbia (L)	Outflow (inconclusive)	10	Y (colony relocation)	California Current	Lyons (2010)

^**1**^ **Life stage:** Br, breeding; NBr, non‐breeding; NS, not specified. Br & NBr refers to data combined from the same species across life stages; Br, NBr refers to data from different species, each in a distinct life stage.

^**2**^ **River name and class:** name of rivers involved in the study, in ascending order of flow according to the framework described by Meijer *et al*. (2021); SUR, several unidentified rivers; BSUR, bay with several unidentified rivers; VS, very small (<10 m^3^ s^−1^); S, small (10–100 m^3^ s^−1^); M, medium (100–1000 m^3^ s^−1^); L, large (1000–10,000 m^3^ s^−1^); VL, very large (>10,000 m^3^ s^−1^).

^**3**^ **River metric** (the most informative as reported by the authors): refers to the specific river effect indicated in the first column of this table; for the overall most informative metric in the paper see Table S3. Unless stated otherwise, river metrics were considered to provide conclusive evidence of river effects. Metrics were classified as inconclusive when evidence for river effects was unclear, not directly tested, or not explicitly interpreted. “Not significant” indicates a reported lack of statistical effect. When multiple metrics were available, the most conclusive one was selected, even if the result was not significant. Distance, distance from colony/seabird to river; Number/control, number of rivers >0 *vs* 0; Number/weighted, treatment of the ‘number of rivers’ metric, in which simple counts are adjusted by river size; Outflow, annual, average, or daily discharge; Plume/non‐river‐specific variables (in alphabetical order), ocean water properties used to characterise plume waters and link river metrics with seabird responses. Chl‐a, chlorophyll‐a concentration; SSS, sea surface salinity; SST, sea surface temperature; Turb, turbidity; Salinity class/zones, fixed areas defined by salinity.

^**4**^ **Study duration (years):** reflects the number of calendar years involved in the research. Multiple values indicate separate periods/data sets; semicolons represent non‐consecutive periods of data collection and hyphens correspond to a reported range.

^**5**^ **Extrinsic disturbances (Y/N):** Y corresponds to studies reporting extrinsic events, namely storms/droughts/floods outside average seasonal thresholds, or specific anthropogenic actions affecting river or seabird systems corresponds during the study period. N corresponds to study conditions of intrinsic hydrologic variability without any mention of extrinsic events. For pollutants, intrinsic variability was assumed unless exacerbated by extrinsic factors (e.g. dam collapse).

^**6**^ **Large Marine Ecosystem:** location of the study within the framework defining the boundaries of the world's 66 Large Marine Ecosystems (LMEs) based on ecological criteria including bathymetry, hydrography, productivity, and trophic linkages (Sherman & Hamukuaya, 2016).

Metrics were classified as *not significant* when tested statistically and reported by the authors as having no significant effect on seabird responses.

When multiple river‐related or plume‐related variables were tested, we identified the metric(s) the authors presented as the most informative for assessing the river's influence on seabirds (Tables 2 and S2). If no variable in a study met this criterion, we selected the least ambiguous option available, even if it was ultimately classified as inconclusive or not significant. In such cases, the inconclusive classification indicates that no other variable in the study provided stronger or more robust evidence. Although evidence strength was assessed for all metrics, the designation *conclusive* was applied only to the metric identified as most informative within each study, linking metric performance directly to both informativeness and strength of evidence. This approach ensured that metric performance was assessed consistently across studies, with each study contributing a single, comparable data point (informativeness: 51 entries), while maintaining transparency about the strength and nature of the reported evidence among eligible entries (conclusiveness: ≤51 entries). By contrast, metric prevalence captured all metrics analysed across studies, regardless of whether they were selected as the most informative (>51 entries in total).

### (5) Seabird responses to river influence (river effects)

Four categories of river effects were created to group studies analysing similar seabird responses (Tables 2 and S2). This categorisation, based on Burger (2023), included proximate effects (habitat selection, diet/feeding behaviour, health and disease) and cascading effects (demographics) of climate change on seabirds, while also ensuring comparability in objectives and methodologies: (*i*) *habitat selection* encompassed spatial ecology studies using seabird density/distribution to infer habitat preferences (Northrup *et al*., 2022); (*ii*) *diet/feeding behaviour* focused on studying seabird prey composition and adaptive foraging strategies through direct biological or behavioural evidence from consumers (Shealer, 2002); (*iii*) *health and disease* addressed impacts on physical fitness and contaminant/pollutant/pathogen exposure (Sebastiano *et al*., 2022); and (*iv*) *demographics* included research on breeding phenology, reproductive success, and survival rates to understand population dynamics (Kendall *et al*., 2009).

### (6) Data analysis

To explore temporal trends and the level of taxonomic resolution in the field, we analysed publication patterns from 2020 onwards and compared them with earlier studies (pre‐2020). Multi‐species studies were disaggregated into single‐species records to allow assessment of both species‐specific and life‐stage‐specific responses to rivers, resulting in a total of 115 seabird–river observations (see Table S4).

Although a formal meta‐analysis was not feasible because of the limited number of studies employing standardised measurements of both seabird responses and explanatory variables across the defined river effect categories, we qualitatively reviewed 10 studies that explicitly tested the relationship between river outflow (or related proxies) and seabird demographic outcomes (see Table S5). For each case, we recorded whether the relationship between river flow and demographic response was described as direct (e.g. increased flow was associated with higher breeding success) or inverse.

We also reviewed studies addressing extrinsic stressors such as marine heatwaves, storms, and large‐scale pollution events, and evaluated whether rivers were interpreted by the study authors as mitigating, amplifying, or exerting mixed influence on seabird responses. Although this extended beyond the foraging ecology focus of this review, it was considered relevant for understanding the broader ecological role of river systems.

Descriptive summaries were produced using Microsoft Excel and RStudio (v. 4.2.2), including frequency counts and cross‐tabulations by environmental variable, taxonomic group, and study characteristics. Visualisations included a PRISMA flowchart, a data‐extraction workflow schematic, a global map of study range/locations, descriptive bar charts contextualising river classes and summarising environmental variable frequencies, and an area‐proportional Venn‐style diagram showing overlap of river effects across studies. These were produced using Excel, base R and *ggplot2* with layout refinements in *GIMP* (v. 2.10.34). No inferential statistical models were applied; publication trends were summarised using frequency distributions. All percentages were rounded to the nearest whole number.

We used artificial intelligence tools to improve the fluency of sentences in British English and to locate inconsistencies in tables (ChatGPT, https://chatgpt.com; NotebookLM, https://notebooklm.google.com).

## III. RESULTS

Our search, screening, and eligibility criteria yielded a total of 51 papers investigating river effects on seabird foraging ecology. Table 2 presents a structured overview of these studies, organised by taxon and including the most informative river metrics used to explore this relationship. Of the 51 papers reviewed, 45 presented conclusive evidence that river‐related variables influenced at least one seabird response, while only six studies failed to detect such effects with the metrics employed (Table S3).

The sections that follow describe the scope and distribution of the reviewed literature, the seabird taxa and life stages represented, the types of environmental variables examined, the performance of river metrics as evidence, and the ways river influences were assessed across seabird response categories.

### (1) Literature review scope and overview

**Fig. 3:**
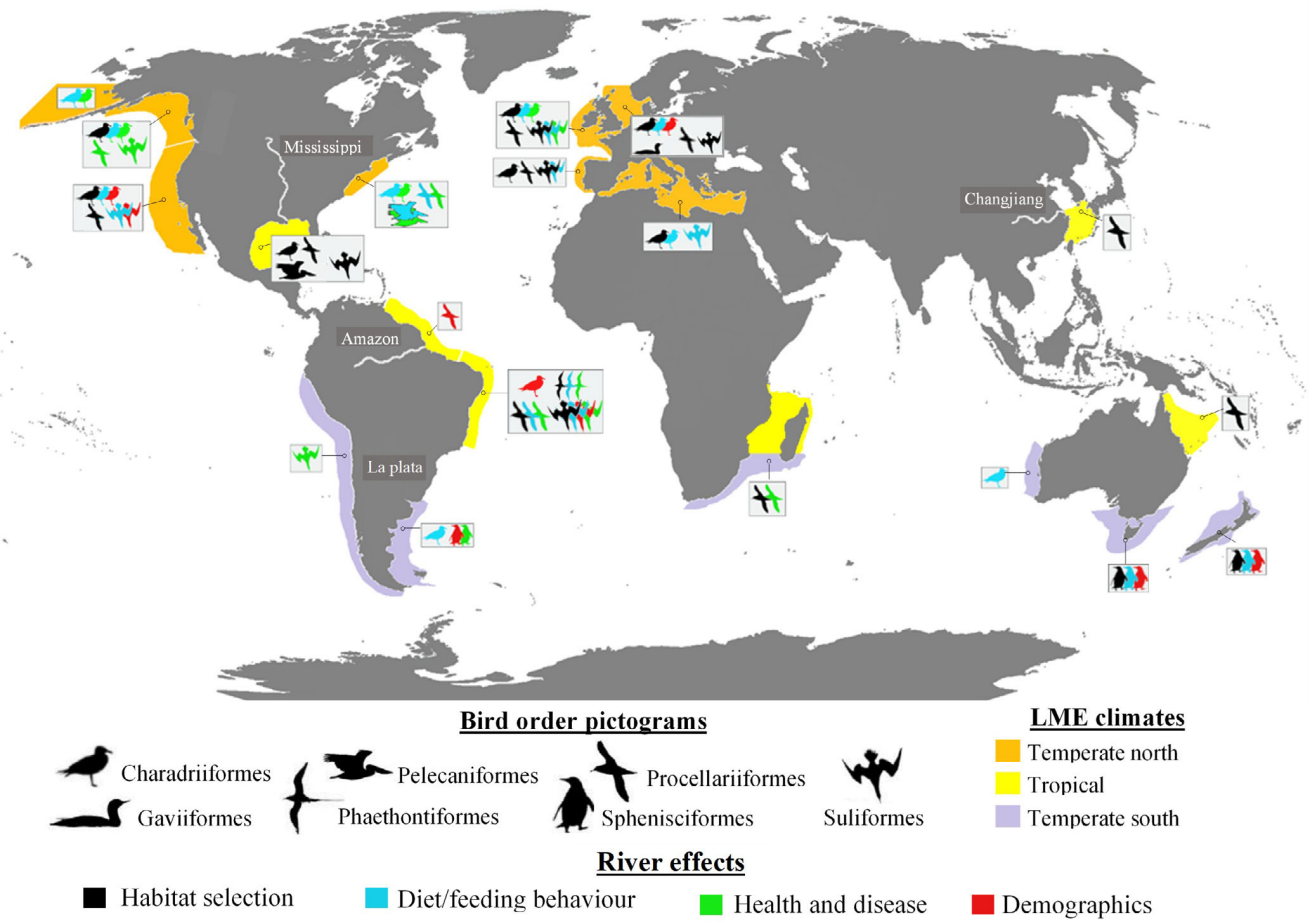
World map of the Large Marine Ecosystems (LMEs) and corresponding river–seabird research. The range of river effects studied by seabird order in various LME climate regions is depicted. Very large rivers (>10,000 m^3^ s^−1^) are also shown.

The 51 reviewed papers spanned 19 of the world's 66 LMEs, reflecting a broad but uneven geographic distribution of research efforts in this topic (Fig. 3); one paper (Day *et al*., 2012) covered two LMEs, yielding 52 LME occurrences for percentage calculations. The California Current and Gulf of Alaska together accounted for 37% of studies, with 13 and six papers respectively. The Celtic Biscay Shelf and North Sea each had four papers, together comprising 15% of the total. The Patagonian Shelf, Iberian Coast, Mediterranean Sea, and South East Australian Shelf each contributed three papers, representing 23% combined. The Gulf of Mexico and East Brazil Shelf had two papers each. The remaining 9 LMEs, each with one paper, collectively made up 17% of the studies (Tables 2 and S4).

Most papers (76%, 39/51) did not involve environmental disturbances, representing baseline studies of river–seabird relationships (Ummenhofer & Meehl, 2017). The remaining 24% (12/51) reported on extreme environmental variations due to extrinsic factors such as anthropogenic disturbances (e.g. dam collapse, colony relocation, establishment of an industrial fishery), as well as intense or sustained climatic anomalies (e.g. storms, marine heatwaves, floods, and droughts outside normal limits) (Table 2). Based on the publication dates of the included papers, since 2020, the proportion reporting extrinsic events increased from 19% (7/36) to 33% (5/15); this may indicate growing interest in the impacts of climate extremes and anthropogenic pressures on aquatic ecosystems and seabird populations.

Study durations ranged from 11 days (Waggitt, Torres & Fraser, 2020) to 68 years (Yen, Huettmann & Cooke, 2004). We identified 32 studies lasting 4 years or less (63%, short term), 13 lasting between five and 20 years (25%, medium term), and six investigating river effects for more than 20 years (12%, long term). Short‐term studies averaged approximately 2 years in duration. Seabird species studied over a period of 20 years or more included the Audubon's shearwater (*Puffinus lherminieri*) (Precheur *et al*., 2016), the black‐legged kittiwake (*Rissa tridactyla*) (Scott *et al*., 2006), the common guilemot/common murre (*Uria aalge*) (Scott *et al*., 2006) (herein referred to as common murre), the little penguin (*Eudyptula minor*) (Colombelli‐Négrel *et al*., 2022), the Magellanic penguin (*Spheniscus magellanicus*) (Rebstock & Boersma, 2018; Clark‐Wolf *et al*., 2023), and the marbled murrelet (*Brachyramphus marmoratus*) (Yen *et al*., 2004).

**Fig. 4:**
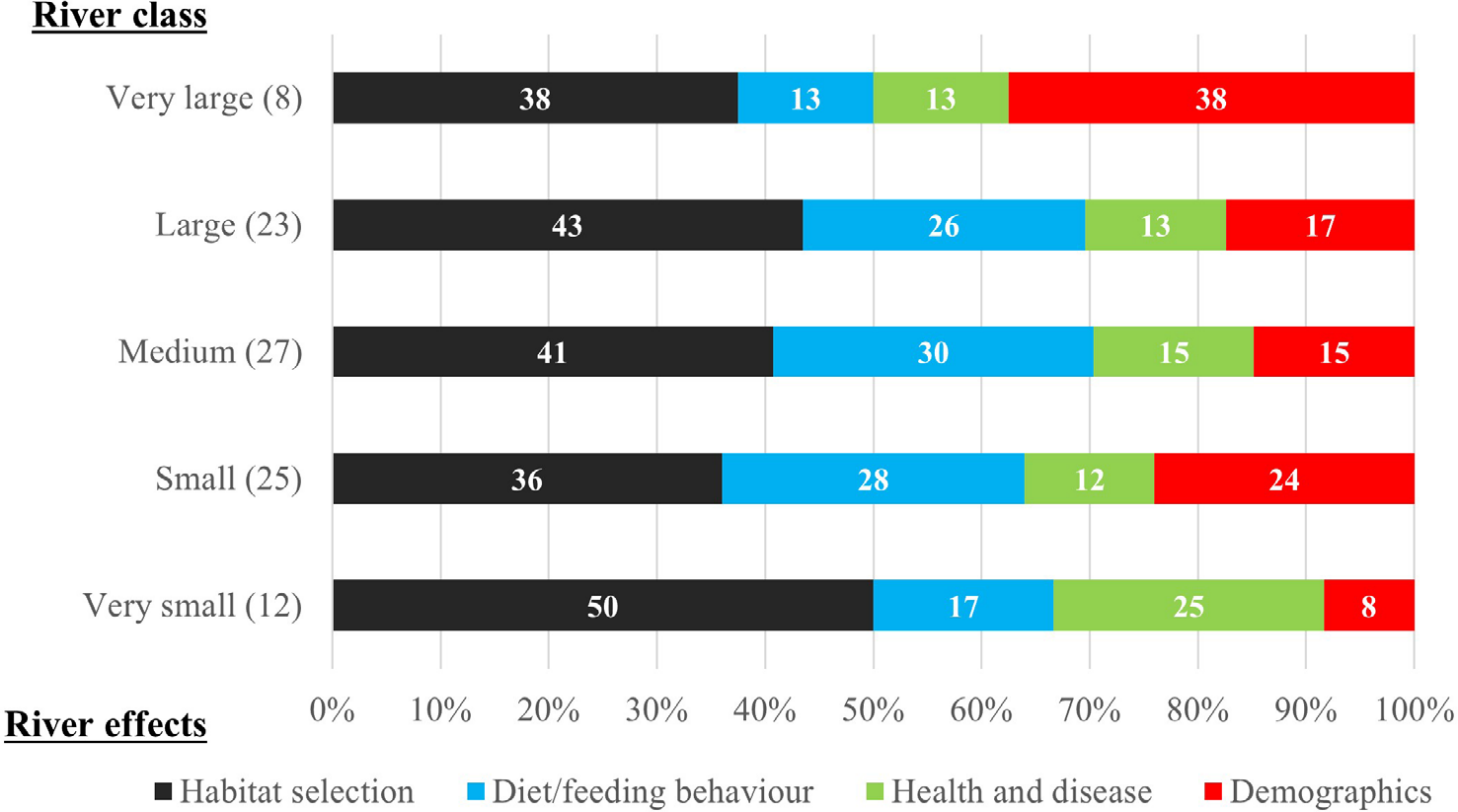
Percentage distribution of the studied river effects in relation to river outflow class (Meijer *et al*., 2021) for all river classes covered in the 51 papers (*N* = 95): very small (<10 m^3^ s^−1^), small (10–100 m^3^ s^−1^), medium (100–1,000 m^3^ s^−1^), large (1,000–10,000 m^3^ s^−1^) and very large (>10,000 m^3^ s^−1^). Different rivers belonging to the same class and studied for the same river effect were counted as a single occurrence of that river class, leading to a total of 95 river, stream or creek occurrences.

The majority of studies (76%, 39/51) focused on a single river class, whereas the remaining 24% (12/51) encompassed multiple river classes (Tables 2 and S2). To disentangle multiple‐river studies, in each paper, different rivers belonging to the same class and studied for the same river effect were counted as a single occurrence of that river class, leading to a total of 95 river, stream or creek occurrences (Fig. 4). Medium rivers were the most commonly studied (27/95 entries) and very large rivers the least (8/95), with the La Plata river appearing most frequently for the ‘very large’ river class category (four papers) (Table S2). Habitat selection was the most common river effect across all river classes (36–50%). Demographic responses were particularly frequent in studies on very large rivers (38%), while health and disease‐related responses were more often associated with studies on very small rivers (25%). Diet/feeding behaviour responses were most frequently associated with intermediate river classes (26, 28, 30%) and least common at the extremes (13 and 17%) (Fig. 4).

The most studied river, the Columbia river, which discharges into the California Current (the most studied LME), was featured in 10 papers. The influence of this large river was examined in the foraging ecology of five seabird species: the Caspian tern (*Hydroprogne caspia*), common murre, double‐crested cormorant (*Nannopterum auritum*), marbled murrelet, and sooty shearwater (*Ardenna grisea*) (Table 2).

Overall, while the California Current emerged as a research hotspot for studies on river influence on seabirds, many important seabird regions around the world remain understudied. Notably, only one publication came from African coasts (Thibault *et al*., 2024) and one from Asia (Matsumoto *et al*., 2016) (Fig. 3). Moreover, although the full range of river effects was reported from all climate regions, diet studies were particularly underrepresented in the tropics, with all evidence originating from a single study in the East Brazil Shelf (Table S4).

### (2) General research characteristics: focal species

In this review 62 species from 12 families were investigated, representing all seven seabird orders across 32 single‐ (63%, 32/51) and 19 multiple‐species (37%, 19/51) papers. Seabirds from the order Charadriiformes were the most studied species (53%, 33/62), followed by Procellariiformes (21%, 13/62) and Suliformes (15%, 9/62). Three orders were equally represented: Gaviiformes (3%, 2/62), Phaethontiformes (3%, 2/62), and Sphenisciformes (3%, 2/62) (Table S4). The least represented order was Pelecaniformes (2%, 1/62).

The most frequently studied species was the common murre, appearing in 10 papers, followed by the marbled murrelet with six papers and the common tern (*Sterna hirundo*) with five. Three species, the herring gull (*Larus argentatus argentatus* and *Larus argentatus smithosianus*), little penguin and sooty shearwater, each appeared in four papers, while the Caspian tern, double‐crested cormorant, great cormorant (*Phalacrocorax carbo*), northern gannet (*Morus bassanus*) and Sandwich tern (*Thalasseus sandvicensis*) were each studied in three. Sixteen species were investigated twice, and the remaining 35 species were represented only once (Table S4).

Most of the 51 papers focused on seabird responses to rivers during the breeding period (28/51, including two studies on eggs; Table S2). Seven studies examined birds during non‐breeding life stages (one involving deceased juveniles), nine did not specify life stage, and seven papers addressed both breeding and non‐breeding periods. Among the papers covering both periods, four followed the same species across life stages, namely Audubon's shearwater (Precheur *et al*., 2016), brown pelican (*Pelecanus occidentalis*; Lamb, Satgé & Jodice, 2020), little penguin (Colombelli‐Négrel *et al*., 2022), and Magellanic penguin (Clark‐Wolf *et al*., 2023), while three papers reported on two species, specifically the common murre and sooty shearwater, with each represented at a different life stage (Zamon, Phillips & Guy, 2014; Phillips, Horne & Zamon, 2017; Phillips *et al*., 2018) (Tables 2 and S2).

In the nine cases where life stage was not specified, this omission likely reflects either the nature of the sampling methods or the broader scope of the study objectives. Two of them analysed regurgitated pellets (Dias *et al*., 2012; Díaz‐Santibáñez, Clark & Zavalaga, 2023), one relied on aerial survey data (Cama *et al*., 2012), and two used opportunistic sampling from dead birds (Bearhop *et al*., 1999; Tavares, Fulgencio de Moura & Siciliano, 2016). The remaining four studies adopted a broad sampling strategy rather than targeting breeding‐specific foraging, aiming instead to assess ecological interactions or threats at the population or community level (Phillips, Horne & Zamon, 2021; Nunes *et al*., 2022; Ribic *et al*., 1997; Masiá, Ardura & Garcia‐Vazquez, 2019).

Research on tropical‐breeding species accounted for 24% of all studied species (15/62 species; Table S4). Studies conducted in tropical ecosystems spanned five LMEs, the Gulf of Mexico, East Brazil Shelf, East China Sea, North Brazil Shelf and Northeast Australian Shelf, as well as the climatically mixed Agulhas Current LME, and comprised eight publications (Ribic *et al*., 1997; Matsumoto *et al*., 2016; Precheur *et al*., 2016; Tavares *et al*., 2016; McDuie, Weeks & Congdon, 2018; Lamb *et al*., 2020; Nunes *et al*., 2022, Thibault *et al*., 2024) (Tables 2 and S4). Several of these studies included non‐tropical or broadly distributed species despite being conducted in tropical regions. The fact that 76% of studied species were non‐tropical breeders should not be interpreted as evidence that rivers affect birds breeding in temperate climates more than those in tropical ones, but instead likely reflect global trends in research effort and development funding (Higgins *et al*., 2006). Across all regions, bird pictograms shown in Fig. 3 represent the range of river effects examined per order rather than the number of independent studies.

Pelagic species were studied nearly as often as coastal ones (57/115 and 58/115, respectively), based on the 115 single‐species observations extracted, agreeing with the possibility that river effects may propagate beyond coastal zones to create offshore foraging opportunities – although this pattern also reflects the scope of available studies. Pursuit diving was the most frequently represented foraging technique overall (41%, 47/115), followed by surface feeding (30%, 35/115) and plunge diving (29%, 33/115) (Table S4; Shealer, 2002). The higher representation of this foraging technique was largely driven by pelagic species, among which pursuit diving predominated, suggesting that specialisation on deeper prey may be associated with vertical plume fronts (Dias *et al*., 2012). By contrast, featured coastal species encompassed a more even distribution of feeding strategies, consistent with behavioural flexibility suited to dynamic estuarine habitats.

According to the IUCN *Red List* (IUCN, 2025), the studied seabird species of conservation concern include: Balearic shearwater (*Puffinus mauretanicus*), listed as Critically Endangered; Barau's petrel and marbled murrelet, both classified as Endangered; and fairy tern (*Sternula nereis*), Leach's storm‐petrel (*Hydrobates leucorhous*), Trindade petrel (*Pterodroma arminjoniana*), and black‐legged kittiwake, all listed as Vulnerable. The guanay cormorant (*Leucocarbo bougainvilliorum*), sooty shearwater and streaked shearwater (*Calonectris leucomelas*) were labelled as Near Threatened. The remaining 52 species were considered of Least Concern. The prevalence of species of conservation concern in the selected studies thus was 16% (10/62; Table S4), diverging from the global estimate of 84% of seabirds facing continuing threats (Dias *et al*., 2019b). This discrepancy underscores that the reviewed studies originated primarily from research on focal species, selected for their indicative value in reflecting essential habitat conditions, irrespective of their conservation status.

### (3) Environmental variables: meteorological and prey abundance indices

**Fig. 5:**
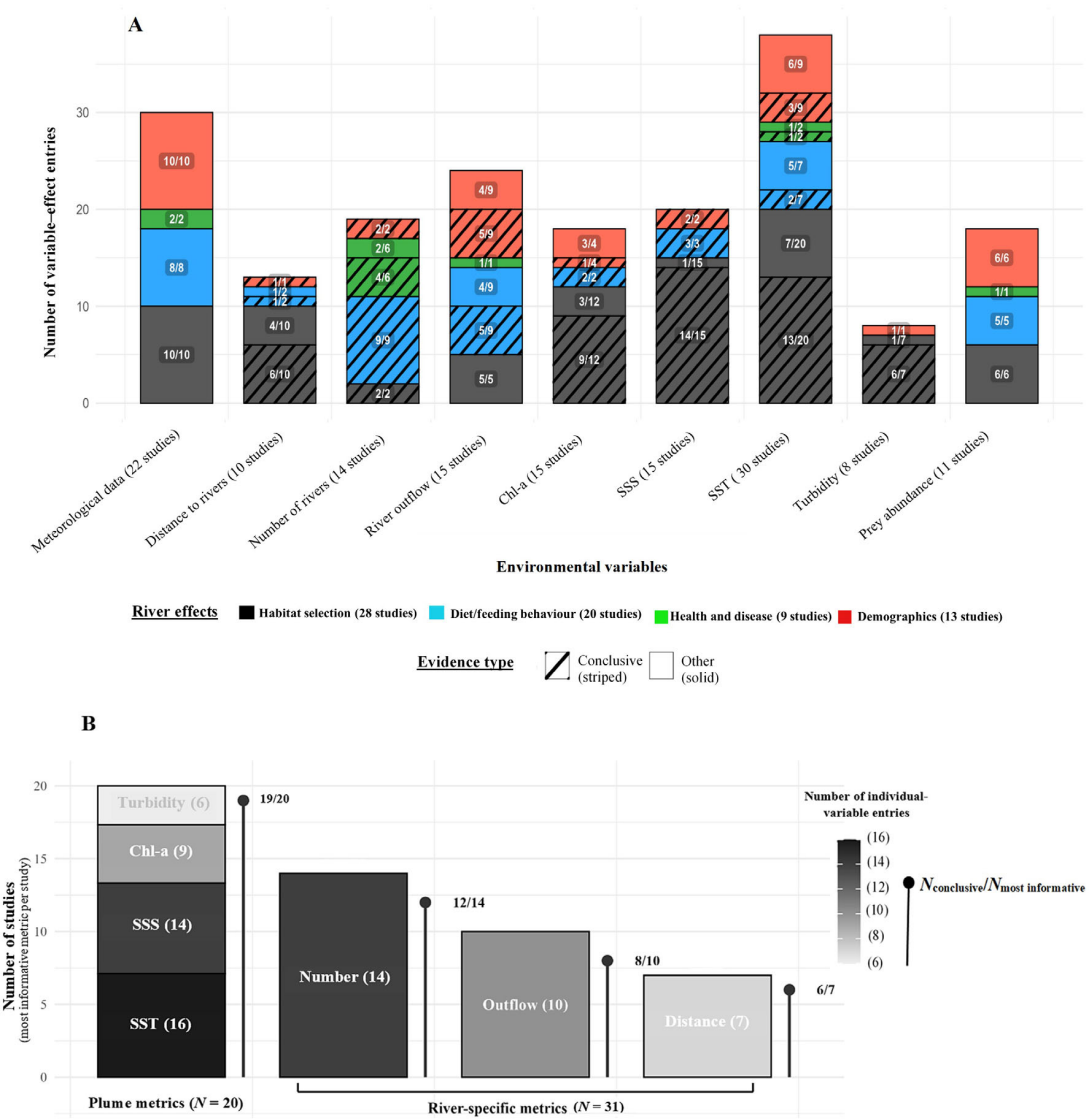
Prevalence and performance of environmental variables used to assess river effects on seabirds. (A) Frequency of the nine extracted environmental variables used across the 51 reviewed studies. Bars show how often each variable was used to assess the four river‐effect categories, accounting for 188 variable–effect entries (total prevalence of variables), as many studies assessed multiple variables and effects. Striped segments indicate entries where the variable was identified as the *most informative* and provided *conclusive* evidence; solid segments represent all other outcomes (inconclusive, non‐significant, or unclassified). Numerical labels show the count of conclusive or ‘other’ outcomes relative to the total entries for each variable–effect combination. (B) Performance and relative frequency of metrics identified as the most informative in each paper (*N* = 51). The figure contrasts plume metrics (*N* = 20) grouped on the left with river‐specific metrics (*N* = 31) grouped on the right. The plume‐metric bar is divided by sub‐metrics (Chl‐a, chlorophyll‐a concentration; SSS, sea‐surface salinity; SST, sea‐surface temperature; turbidity), with shading intensity reflecting their relative frequency (individual‐variable entries for plume indicators are not additive because several authors identified combinations of plume variables as most informative). Points and vertical lines (lollipops) indicate the proportion of studies providing conclusive evidence for each most‐informative metric.

Meteorological variables were reported in 22 of the 51 reviewed studies and spanned global, regional, and local scales (Fig. 5A; Table S2). Because individual studies often incorporated multiple environmental variables across different spatial scales, the frequencies reported below are not mutually exclusive and therefore are not additive. Among global climate indices, the El Niño Southern Oscillation (ENSO) indicators such as the Southern Oscillation Index (SOI), Oceanic Niño Index (ONI), and Multivariate ENSO Index (MEI) were the most frequently reported, appearing in seven studies. Other global indices included the Pacific Decadal Oscillation (PDO, four studies), the North Pacific Gyre Oscillation (NPGO, two), the North Atlantic Oscillation (NAO, one), and the Southern Annular Mode (SAM, one). Regional drivers included upwelling indicators such as the Biologically Effective Upwelling Transport Index (BEUTI), the Multivariate Ocean Climate Index (MOCI), and other physically driven upwelling indices (e.g. daily upwelling anomalies), reported collectively in seven studies. Local meteorological variables such as wind (eight studies), rainfall (seven), tidal currents or tidal action (six), air temperature (one), and wave height or sea state (two) were included in 16 studies. These local variables were typically used to characterise immediate environmental conditions at study sites and were often embedded within physical or oceanographic models, particularly wind, limiting the ability to isolate individual effects. Despite their potential importance in modulating river flow and plume location/structure, environmental variables were omitted entirely in 29 studies (57%), highlighting an underuse of weather‐related and other physical drivers in river–seabird ecological analyses.

Most studies did not provide quantitative data on prey abundance (40/51, 78%). Those that did (11 studies), quantified prey using trawl catches (six studies), acoustic equipment (two), a combination of both methods (two) or did not specify the fish survey method (one) (Table S2).

Prey abundance data were used to examine habitat selection responses, assessing whether areas of high seabird densities coincided with increased prey availability (six studies: Anderson *et al*., 2007; Haynes *et al*., 2011; Kowalczyk *et al*., 2015a; Phillips *et al*., 2017; Pastran, Drever & Lank, 2021; Phillips *et al*., 2021). Prey abundance was also used to explore potential causal relationships between river influence and seabird diet composition (five studies: Anderson *et al*., 2007; Collar, Roby & Lyons, 2017; Kowalczyk *et al*., 2015a; Lyons, 2010; Lyons *et al*., 2014), demographic responses (six studies: Anderson *et al*., 2007; Kowalczyk *et al*., 2015a; Collar *et al*., 2017; Rebstock & Boersma, 2018; Colombelli‐Négrel *et al*., 2022; Lyons, 2010), and health and disease outcomes (one study; Rebstock & Boersma, 2018) (Fig. 5A; Table S2).

### (4) River metrics and evidence: prevalence and performance

Research varied in the metrics used to assess river influence on seabirds, allowing comparison of their prevalence and performance (Fig. 5) (Section II.4).

Regarding metric prevalence, studies more frequently employed plume indicators, particularly SST (30 studies), and to a lesser extent SSS (15) and Chl‐a (15), than river‐specific metrics, such as river outflow (15 studies), number of rivers (14 studies), and distance to river mouths (10) (Fig. 5A). While plume indicators were widely used, often in combination, turbidity was the least‐studied variable related to river influence (8/51 studies). These frequencies are not additive in terms of total study count, because several studies applied multiple river‐related metrics simultaneously (Fig. 5A; Tables S2 and S3).

To compare metric performance, for each paper we allocated a single river‐related metric considered most informative by the authors (Table S3). Across the 51 reviewed papers, river‐specific metrics were more frequently selected as the most informative (61%, 31/51) than plume indicators (39%, 20/51) (Fig. 5B). This pattern was also evident in studies that applied both plume indicators and river‐specific metrics within the same analysis (Table S2), suggesting that river‐specific variables more often provided the clearest explanatory links between freshwater inputs and seabird responses, consistent with their inherent specificity.

When plume indicators were considered the most informative (20/51 studies), often relying on combinations of SST, SSS, Chl‐a and turbidity measurements, 95% (19/20) yielded conclusive evidence (exception: Nunes *et al*., 2022) (Fig. 5B; Table S3). On the other hand, studies with river‐specific metrics as most informative showed lower overall conclusiveness (84%, 26/31), comprising 12 of 14 studies using number of rivers, eight of 10 using river outflow, and six of seven using distance to river mouths (Fig. 5B; Table S3). Together, these findings suggest that different metrics have complementary strengths, highlighting the value of combining river‐specific and plume variables to provide evidence of river effects globally.

Although a high proportion of the reviewed studies generated conclusive evidence using their respective metrics (45/51; 19 plume, 12 number of rivers, eight river outflow, and six distance to river mouths; Fig. 5B), synthesising quantitative evidence across systems remained challenging due to a lack of standardisation in how river‐related variables were defined, measured, and reported. For instance, ‘plume’ was described variously as static salinity‐defined zones (e.g. Anderson *et al*., 2007), gradients of remotely sensed temperature anomalies (e.g. Rebstock & Boersma 2018; Clark‐Wolf *et al*., 2023), or as plume area (e.g. Zamon *et al*., 2014; Phillips *et al*., 2018). River outflow was quantified at different temporal resolutions (daily, monthly, annual averages), either as a standalone variable (e.g. Mauco & Favero, 2005; Colombelli‐Négrel *et al*., 2022) or embedded within oceanographic models (e.g. Scott *et al*., 2006; Schwemmer *et al*., 2009) (Table S2).

Similarly, the number of rivers metric often relied on author‐defined habitat categories (e.g. estuarine *versus* marine) that were not always clearly ecologically justified or spatially delineated. During screening, lack of specific contextual information about the studied areas in terms of river influence led to the exclusion of otherwise suitable studies and a consequent reduction in the number of papers available for synthesis (e.g. Santora *et al*., 2014; Clatterbuck *et al*., 2021; Table S1), reflecting a broader challenge of incomplete methodological and contextual reporting also noted by Grant, Bond & Lavers (2022). Among the applications of the number of rivers metric (14/51), two distinct implementation approaches emerged. The most common (11/14) used control‐type designs comparing sites with and without rivers, whereas the remaining three studies used more refined approaches, such as ordinal or weighted river counts. Although the control‐based approach was statistically conclusive in most instances (9/11), it may be susceptible to confounding by unmeasured environmental variables and simplifies river influence as a binary presence/absence factor. By contrast, all three refined applications of the metric yielded conclusive outcomes and were able to capture ecological nuances in riverine influence (Tables 2 and S3).

### (5) Seabird responses to river influence: assessment by response category

**Fig. 6:**
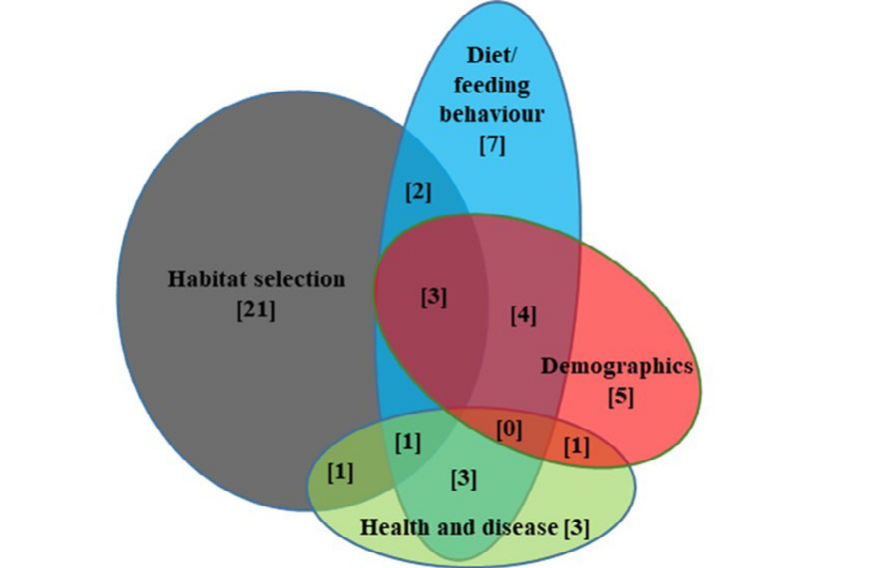
Venn‐style diagram illustrating the four main effects of rivers on seabirds. Number of papers is provided in brackets (*N* = 51 studies); intersections represent studies with multiple river effects.

Seabird responses (i.e. river effects) were unevenly covered across the literature. Of the 51 papers, 36 focused on a single river effect, while 15 addressed more than one. Considering both single‐ and multi‐effect studies, we identified 28 papers on habitat selection, 20 on diet/feeding behaviour, nine on health and disease, and 13 on demographics, resulting in 70 total river effect entries (Table S3; Fig. 6). The lower occurrence of studies on health and disease effects highlighted a persistent gap in understanding the physiological and toxicological pathways linking rivers to seabird populations. Greater integration of health and demographic studies could strengthen causal inference, for example through Directed Acyclic Graph (DAG) frameworks (Franks, Ruxton & Sherratt, 2025). Four papers described three combined river effects (Anderson *et al*., 2007; Kowalczyk *et al*., 2015b; Poupart *et al*., 2017; Nunes *et al*., 2022), but no study simultaneously reported all four potential effects considered in this review (Fig. 6; Table S2).

To illustrate how seabird responses aligned with environmental variables and river‐related metrics, we assessed how often each variable was used for a given effect and how often it provided the most informative and conclusive evidence when applied for that effect (Fig. 5A); since studies analysed multiple variables and effects, the entries are not additive, however, each entry represents one study for a given variable and effect.

Habitat selection was most commonly assessed using SSS and SST, reported in 15 and 20 studies for this response, respectively. Other plume indicators, such as Chl‐a (12 studies) and turbidity (seven) were used less often for this response type. Among river‐specific metrics, distance to river mouth was used in 10 habitat selection studies, with outflow (five) and number of rivers (two) being less represented. The metrics with the highest frequency of conclusive evidence within this response type were number of rivers (2/2), SSS (14/15 studies) and turbidity (6/7) (Fig. 5A; Tables S2 and S3). The influence of turbidity on seabird habitat selection was frequently related to plume location and prey‐accessibility contexts, but quantitative relationships between turbidity and prey‐abundance indices were less thoroughly examined (2/7) (Table S2). Increased spatial co‐occurrence between seabirds and potential prey was reported with increasing plume area and turbidity, suggesting that turbidity may attract marine predators; however, the lack of direct biological or behavioural evidence of prey consumption prevented inference about turbidity effects on diet and feeding behaviour (Phillips *et al*., 2021).

Diet and feeding behaviour responses were most frequently assessed using river‐specific variables, particularly colony comparisons based on number/control approaches (nine studies) and river outflow (nine). The number of rivers metric yielded conclusive results in all diet studies where it was applied (9/9), whereas river outflow was conclusive in five of nine cases. Plume indicators, such as Chl‐a (two studies) and SSS (three), were used less frequently but were conclusive in all cases. SST was used in seven diet studies but was conclusive in only two. There were no diet studies using turbidity (Fig. 5A).

Health and disease effects were reported using the number of rivers in a given area (six studies), SST (two) and river outflow (one), yielding conclusive evidence in four out of six studies for number and in one out of two studies for SST. River outflow did not yield conclusive results for this effect (Fig. 5A). River effects on health and disease were linked to physiological responses or pollution pathways, respectively. River influence on contaminant loads was described in eight cases (8/9; Elliott *et al*., 1989; Day *et al*., 2012; Masiá *et al*., 2019; Robuck *et al*., 2020; Nunes *et al*., 2022; Binkowski *et al*., 2021; Diaz‐Santibañez *et al*., 2023; Thibault *et al*., 2024), while physiological responses (e.g. changes in body condition or contaminant‐related effects on liver lipid reserves) were reported less frequently (2/9; Rebstock & Boersma, 2018; Robuck *et al*., 2020) and remain underexplored.

Demographic responses were detected using SST (nine studies), river outflow (nine), Chl‐a (four), SSS (two), number of rivers (two), turbidity (one) and distance to rivers (one). River outflow produced conclusive demographic evidence more often (5/9) than SST (3/9). Chl‐a (1/4) yielded the lowest proportion of conclusive evidence. Research on demographic responses (13 studies) more frequently incorporated meteorological (10/13) and prey‐abundance variables (6/13) than habitat‐selection studies (10/28 and 6/28, respectively), likely to support or contextualise interpretations on population dynamics (Fig. 5A; Table S2).

Data to investigate relationships between outflow/plume strength variations and seabird demographic responses were available in 10 studies (Table S5), using historic mean monthly outflow. Visual inspection suggested a pattern: five studies of rivers with mean monthly outflows above or equal to 1,000 m^3^ s^−1^ reported inverse relationships between outflow and seabird demographics, whereas four studies of rivers with mean monthly outflows below 1,000 m^3^ s^−1^ reported direct positive relationships. The Amazon–Orinoco system represented an exception to this pattern, consistent with its atypical scale and hydrology. Nevertheless, the limited data set prevented the use of a generalised linear model, hindering our ability to derive statistically supported conclusions. In addition, there was considerable mismatch in study duration and spatial scale among the selected studies, which is problematic when attempting to integrate demographic responses from ecologically different systems operating on different temporal scales (Orgeret *et al*., 2022).

Overall, the effectiveness of a metric can depend on the type of seabird response under investigation. Plume‐based metrics, especially SSS and SST, were most effective for explaining habitat selection, while river‐specific variables, particularly number/control‐based metrics, were perhaps more useful for detecting effects on diet and health. Outflow may be particularly relevant for demographic studies. The findings emphasise the importance of aligning environmental metrics with clearly defined ecological questions when assessing the influence of rivers on seabirds.

## IV. DISCUSSION

Our review demonstrates that rivers exert a pervasive influence on seabird ecology, with approximately 88% (45/51) of studies providing clear evidence of river effects across diverse taxa, life cycle stages, and foraging strategies. We conclude that rivers and their plumes are critical coastal features shaping seabird habitat use, diet, health, and demographic outcomes. The diverse ways in which seabirds interact with riverine and plume systems, combined with the wide range of study designs and metrics employed, underscores both the ecological importance of these influences and the challenges inherent in assessing them comprehensively.

While some studies suggested that the primary influence of terrestrial outflows is on predator–prey interactions (Phillips *et al*., 2017; Colombelli‐Négrel *et al*., 2022), investigating the influence of rivers on seabirds through prey abundance indices proved challenging. The inclusion of ocean‐based prey data in river–seabird relationship studies likely was limited by the expense and difficulty of obtaining data from at‐sea surveys. However, despite some studies indicating that river discharge impacts on forage fish availability can affect breeding success (Kowalczyk *et al*., 2015a; Collar *et al*., 2017; Rebstock & Boersma, 2018; Colombelli‐Négrel *et al*., 2022), seabird distribution (Pastran *et al*., 2021), and diet (Lyons *et al*., 2014), the relationship between fish abundance, river/plume input, and seabird behaviour was not always clear, likely due to several confounding factors (Haynes *et al*., 2011; Phillips *et al*., 2017). Indeed, seabird responses may be influenced by threshold levels of prey abundance (Ballance, Ainley & Hunt Jr, 2001), which vary due to physical cues/environmental conditions (Hunt *et al*., 1999), inter‐ and intraspecific competition, colony size (Ronconi & Burger, 2011), or reproductive demands (Haynes *et al*., 2011). In this context, environmental conditions, particularly river discharge and large‐scale climate indices that modulate freshwater flows through effects on precipitation and runoff may play a dominant role in explaining seabird responses to varying prey abundance. These factors can outweigh others, such as colony size, by influencing both the relative abundance of specific prey and the capability of seabirds to locate them (Lyons *et al*., 2014). This interpretation is consistent with studies where seabirds from nearby colonies, exposed to differing inferred levels of river influence, showed different responses to fluctuations in prey abundance (Litzow & Piatt, 2003; Hostetter *et al*., 2023). A river influence on the availability/accessibility of specific prey, such as lipid‐rich schooling fish species, may enable seabirds to allocate time more flexibly and allow consistent food delivery to offspring regardless of prey abundance (Golet *et al*., 2000; Litzow & Piatt, 2003). Repeated surveys emphasised that prey detectability and accessibility (i.e. the time taken to locate, capture, and eat prey), which involves other potential river‐associated environmental variables, such as water depth or turbidity, could be more reliable predictors of predator responses than prey abundance itself (de la Cruz *et al*., 2021).

### (1) River effects on seabird habitat selection

The effects of rivers on seabird habitat selection were examined using statistical models that correlate seabird spatial distribution data obtained from ship‐based surveys, aerial observations, or tracking, with environmental variables from coastal regions influenced by rivers. By integrating river‐specific and non‐river‐specific predictors, these models evaluated the relative importance of terrestrial/riverine *versus* marine habitat features on seabird distribution (Raphael *et al*., 2015). Flow data were rarely used in these models, possibly due to the stochastic nature of flow across regions, data scarcity for small rivers (due to low numbers of gauging stations; The Ad Hoc Group *et al*., 2001), and the prioritisation of historically significant oceanographic variables (Ainley *et al*., 2012). Some studies incorporated flow information indirectly through hydrodynamic modelling (Schwemmer *et al*., 2009; Phillips *et al*., 2018; Waggitt *et al*., 2020), although such approaches require intensive computational resources and data access (Phillips *et al*., 2018).

By contrast, models using straightforward predictors such as distance to river mouths, often adjusted by river size, have been effective in capturing seabird spatial responses and revealing their behaviour relative to these water bodies (Garcia‐Heras *et al*., 2024). While some authors used stream order (Strahler scale) to define the size of the rivers of interest (Barrett, 2008; Haynes *et al*., 2011), others applied an arbitrary outflow threshold to distinguish major from non‐major rivers in their studied region (Poupart *et al*., 2017). For instance, marbled murrelets in British Columbia and Alaska showed a preference for foraging near stream mouths, favouring estuaries over other coastal areas (Haynes *et al*., 2011), but avoided high flows, calculated as the number of streams weighted by stream order, within a 3 km radius (Yen *et al*., 2004; Barrett, 2008; Pastran *et al*., 2021). Small‐scale rivers offered predictable food sources, while larger rivers mainly served as flyways to inland nesting sites (Barrett, 2008; Haynes *et al*., 2011; Armstrong *et al*., 2020; Pastran *et al*., 2021). The average distance seabirds maintain from rivers during foraging may reflect the extent of the river plume (Auricht *et al*., 2022) and is likely influenced by whether species adopt more onshore/coastal or offshore/pelagic foraging strategies (McDuie *et al*., 2018). However, spatial and seasonal variability in plume extent, often poorly documented, complicates the selection of appropriate spatial scales in modelling (Fredston‐Hermann *et al*., 2016; Auricht *et al*., 2022). This may result in distance‐to‐river variables showing statistically insignificant or weak associations with seabird responses, even when ecological relevance exists (Schwemmer *et al*., 2009; Lamb *et al*., 2020).

Ocean water properties have been frequently used to map seabird spatial responses to river plumes, particularly using frontal structures and gradient slopes to indicate frontal strength (Zamon *et al*., 2014; Rebstock & Boersma, 2018; Clark‐Wolf *et al*., 2023). Riverine fronts, primarily driven by salinity, often exhibit associated gradients in temperature and turbidity, making them visually or thermally detectable by seabirds (Belkin & Cornillon, 2007). Preferences for specific frontal areas appear to depend on both foraging guild (surface, plunge or dive feeders) and the scale and resolution of tracking data (Barrett, 2008; Cumming *et al*., 2012). Diving species such as marbled murrelets and shearwaters may benefit from inner plume fronts of smaller rivers, while surface feeders such as terns or pelicans exploit outer fronts from medium‐to‐large rivers (Garthe, 1997; Skov & Prins, 2001; Phillips *et al*., 2017; de la Cruz *et al*., 2021; Lukies, Gaskin & Whitehead, 2021). These contrasting habitat preferences effectively define functional distances from river mouths, illustrating that the ecological relevance of distance varies with both river magnitude and foraging guild.

Rivers enhance horizontal thermal gradients, attracting both prey and predators (Cama *et al*., 2012; Peck‐Richardson *et al*., 2018; Costa *et al*., 2020), while also creating vertical stratification that generates productive zones (Pastran *et al*., 2021). However, productivity does not always equate to prey abundance, and may instead affect prey behaviour (Waggitt *et al*., 2018). While SSTs can modulate predator–prey relationships due to prey temperature thresholds (Barrett, 2008; Broadley *et al*., 2022), overreliance on this single metric might complicate the interpretation of seabird responses (Boutin *et al*., 2013), as other local factors like seasonal river outflows might play a more significant role in seabird foraging decisions than previously anticipated (Hernández, Oro & Sanz‐Aguilar, 2017). For example, in temperate regions, SST–seabird associations shift in response to changes in runoff timing and reproductive status (Barrett, 2008). In tropical systems, higher SSTs correlated with rainfall‐driven discharge, potentially driving seabird prey availability and survival (Precheur *et al*., 2016).

Persistent river plumes, causing frontal areas and other hydrographic phenomena, have been studied in relation to seabird occurrence in plume‐related salinity fronts (Skov & Prins, 2001; Zamon *et al*., 2014). These dynamic features have led to evaluations of the combined influence of salinity and other environmental variables on seabird foraging activity (Markones, 2007; Kowalczyk *et al*., 2015b; González Carman *et al*., 2016; Lambert *et al*., 2018; Daudt *et al*., 2019). However, depending on the model and input variables, river influence *via* SSS might not be detected due to issues such as collinearity, potentially skewing the importance of some environmental variables (e.g. Chl‐a) for seabird distributions (Daudt *et al*., 2019). Additionally, Chl‐a should be interpreted with caution when assessing impacts on seabirds, not only because it may not be the best predictor, with variables such as distance to the colony and, in some cases, distance to rivers playing a synergistic role in habitat selection (Louzao *et al*., 2011; Afán *et al*., 2021; de la Cruz *et al*., 2021), but also due to uncertain time lags between phytoplankton blooms and effects on higher trophic levels (Precheur *et al*., 2016; Colombelli‐Négrel *et al*., 2022). Lags of 1 year have been described in demographic studies, but analyses with no lag were common in habitat selection studies (Matsumoto *et al*., 2016). Thus, it is essential to analyse other environmental variables in parallel with Chl‐a (Sulc, 2019; Afán *et al*., 2021).

Our review confirms that turbidity is currently overlooked as a dynamic descriptor of oceanic habitats, despite its potential to constrain the foraging abilities of many marine species (Henkel, 2006; Darby *et al*., 2022). While turbid waters may be productive and visually attract some seabirds (Phillips *et al*., 2017, 2021), many other species appear to favour clearer areas for prey capture (McDuie *et al*., 2018; de la Cruz *et al*., 2021). For example, during intense river runoff, little penguins primarily transited through turbid, highly productive waters, with their core foraging areas remaining in less turbid, less‐productive regions (Kowalczyk *et al*., 2015b). Plume‐related turbidity can, therefore, impact habitat use, but with limited quantitative links to feeding behaviour (e.g. prey capture success or dietary shifts), the effect of water clarity on the allocation of foraging strategies remains unclear (Haney & Stone, 1988).

### (2) River effects on diet and feeding behaviour

Freshwater inputs affect prey availability and accessibility, therefore shifts in seabird diets can indicate changes in coastal conditions, including river flow variations (Dias *et al*., 2012; Jodice *et al*., 2019). In Punta Rasa, Argentina, ENSO events affected the diet of common terns, with increased freshwater outflows associated with a time‐lagged decrease in estuarine fish consumption and an increase in marine fish due to bottom‐up controlling forces (Mauco & Favero, 2005). By contrast, diet variability in double‐crested cormorants in the California Current LME, also influenced by large‐scale climate indices linked to ENSO (MEI, NPGO), was regulated by a marine prey intrusion into estuarine areas, which provided a direct alternative forage fish resource (Lyons *et al*., 2014). Seabirds adjusted their diet and reliance on estuarine regions based on environmental fluctuations affecting resource availability (Jones *et al*., 2010; Dias *et al*., 2012; Gladics *et al*., 2015). During ENSO events, associated with marine heatwaves and suboptimal years with warm or weak upwelling leading to low overall fish productivity, clupeoid fish, including anchovies, became critical prey for seabirds (Santora *et al*., 2014). While clupeids do not specifically target riverine areas for spawning, river outflows into estuaries offer favourable conditions, with higher nutrient concentrations and more sheltered conditions than the ocean (Parrish, Nelson & Bakun, 1981).

Seabird diet and variations in riverine input have been studied using prey capture/delivery observations, pellet analysis, and tissue stable isotope signatures from sites along the terrestrial–marine gradient (Bearhop *et al*., 1999; Greenwell *et al*., 2021) or at different distances from major rivers (Poupart *et al*., 2017). Variations in the diet of Australian fairy tern colonies showed that higher riverine discharge, with a subsequent increase in clupeid spawning during the winter months, impacted the diet of marine colonies more than estuarine ones, suggesting the former to be more vulnerable to changes in prey availability and emphasising the increased stability provided by estuarine habitats (Greenwell *et al*., 2021). Control sampling methods highlight coastal areas near major rivers as diverse feeding habitats, enabling seabirds to adapt to environmental variability with sufficient prey choice (Bearhop *et al*., 1999; Litzow & Piatt, 2003; Binkowski *et al*., 2021; Greenwell *et al*., 2021).

Stable isotope data have provided information on resource use, movements, and trophodynamics, with the use of δ^34^S, δ^13^C and δ^15^N, potentially improving assessments of the relative importance of freshwater systems to seabirds (Robuck *et al*., 2020; Binkowski *et al*., 2021; Raoult *et al*., 2024). Further evidence of dietary responses being shaped by freshwater inputs includes great cormorants from marine colonies consistently foraging at inland freshwater sites and exhibiting trophic overlap with freshwater colonies (Farinós‐Celdrán, Robledano‐Aymerich & Palazón‐Ferrando, 2019). Common terns from freshwater colonies showed shorter foraging ranges than those from marine colonies, reflecting more predictable prey availability (Kralj *et al*., 2025). Likewise, little penguin colonies further from rivers relied more on squid and had extended foraging ranges, while those closer to rivers had stable foraging ranges, even during dietary shifts, potentially due to the productivity of nearby river plumes (Poupart *et al*., 2017). Ultimately, seabirds may use freshwater regions either during periods of increased demand or permanently (Bearhop *et al*., 1999). However, utilising freshwater resources or extending foraging ranges to reach these areas may expose seabirds to anthropogenic threats such as conflicts with fluvial fisheries or disruption from dredging activities (Bearhop *et al*., 1999; Mattern, 2020; Mallory *et al*., 2022; Russell *et al*., 2022; Ainley & Wilson, 2023).

### (3) River effects on seabird health and disease

The number of river mouths within seabirds' foraging ranges may contribute to their level of exposure to pollutants (Binkowski *et al*., 2021; Diaz‐Santibañez *et al*., 2023). For example, Guanay cormorant colonies on the coast of Peru with access to more than three rivers exhibited significantly higher plastic contamination levels than colonies close to two, one, or no rivers (Diaz‐Santibañez *et al*., 2023). Conversely, seabirds in the Seine estuary and in an offshore island in the English Channel with no major rivers showed similar mercury contamination in chicks, implying their marine prey were the main mercury source (Binkowski *et al*., 2021). Thus, rivers may contribute to pollutant accumulation in estuaries (Finger *et al*., 2016; Robuck *et al*., 2020; Thorne *et al*., 2021) or offer diverse food resources that potentially reduce bioaccumulation in seabirds (Clatterbuck *et al*., 2021). Some studies show that rivers can act as filters, retaining and modifying materials before they reach the ocean, where dilution and mixing decrease exposure to birds (Emelyanov, 2005). Other studies have indicated that for some pollutants, such as mercury or microplastics, contamination levels can be higher further from the coast due to atmospheric circulation and current effects (Masiá *et al*., 2019; Benemann *et al*., 2024), which may exceed their dispersion through river runoffs into coastal waters (Day *et al*., 2012). The foraging ecology of aquatic birds and the nature of the pollutants [e.g. persistent organic pollutants (POPS), heavy metals, per‐and polyfluoroalkyl substances (PFAS), plastics] can influence whether riverine, estuarine, or offshore marine colonies face increased contamination risk over time (Elliott *et al*., 1989; Suzuki, 2012; Brasso *et al*., 2015; Sherman & Hamukuaya, 2016; Champoux & Boily, 2017; Thibault *et al*., 2024).

The health aspect of river–seabird relationships also is underexplored, with only one study reporting the effects of river plume strength on body condition *stricto sensu* (Rebstock & Boersma, 2018), and another quantifying liver lipid reserves as a proxy of body condition in relation to river‐associated contaminant exposure (Robuck *et al*., 2020). Weaker plumes during winter migration, as measured by the Rio de la Plata plume index, were significantly associated with better body condition and earlier return dates for female Magellanic penguins, likely due to higher prey encounter rates closer to their breeding colonies. This was supported by the fact that females in better body condition and arriving earlier laid larger eggs (Rebstock & Boersma, 2018). Robuck *et al*. (2020) examined liver lipid reserves in wild seabirds from marine and estuarine habitats in relation to PFAS concentrations, but their results were discussed separately for each habitat, limiting direct comparisons and conclusions about an influence of rivers. Their supporting information indicated that seabirds from offshore regions had higher liver lipid reserves and lower perfluorooctane sulfonate (PFOS) levels compared to those from estuarine colonies. However, the lack of baseline data on liver phospholipid levels for their study species restricted the ability to assess the significance of these findings (Robuck *et al*., 2020).

Demonstrating a relationship between body condition and habitat quality amid environmental variability is challenging due to complex interactions between environmental conditions and taxon‐specific characteristics (Phillips, Guilford & Fayet, 2023). Nevertheless, comparing health variables across colonies of the same species within the terrestrial–marine gradient and linking physiological indices with reproductive success could reveal underlying processes, such as nutritional stress from adverse weather, food shortages or contaminant exposure, and help assess the role of rivers amid these stressors (Colombano *et al*., 2022; Sebastiano *et al*., 2022; Vanstreels, Uhart & Work, 2023; Wells *et al*., 2023). Investigation of multiple seabird health indices could enhance their role as aquatic ecosystem sentinels, highlighting sublethal physiological impacts from estuarine influence (Thibault *et al*., 2019; Mallory *et al*., 2022; Burger, 2023).

### (4) River effects on seabird demographics

Seabird demographic variations have been linked to oceanographic variables like SST (Scott *et al*., 2006; Precheur *et al*., 2016; Rebstock & Boersma, 2018) and turbidity (Tavares *et al*., 2016). Increased turbidity can impact prey‐capture rates, leading to nutritional stress or starvation, causing birds to prioritise survival over reproduction and hence to population declines (Lukies *et al*., 2021).

Seabird populations dependent on rivers with lower discharge rates (mean monthly discharges below 1000 m^3^ s^−1^) showed direct relationships of breeding success with river flow, as increased flow improved habitat quality and prey availability (Kowalczyk *et al*., 2015a; Seher *et al*., 2022). By contrast, for high‐volume rivers (mean monthly discharges above 1000 m^3^ s^−1^), the inverse relationship was found, where high flows increased mortality or resulted in declines in fledging success per breeding pair and in the number of breeding pairs (Anderson *et al*., 2007; Tavares *et al*., 2016; Collar *et al*., 2017).

Audubon's shearwaters nesting on an island influenced by the Amazon river showed a slight positive correlation between apparent adult survival rates and river discharge, although with a one‐year lag. However, concurrent management strategies to control terrestrial predators at the study site may have affected the results by contributing to reduced emigration and/or reducing the delayed costs of failed breeding (Precheur *et al*., 2016). This region also is at risk of eutrophication, with higher riverine discharges potentially leading to harmful algal blooms and hypoxia (Deininger & Frigstad, 2019; da Costa *et al*., 2022). No studies have definitively linked rivers to seabird demographics through eutrophication or algal blooms, but several avian mortality events related to algal blooms in estuaries have been described, and circumstantial evidence exists of very large river flows being associated with low habitat quality and low occurrences of seabirds and cetaceans in the tropics (Mondreti *et al*., 2020; Rattner *et al*., 2022).

Our analysis of the literature suggests that river magnitude can influence how seabird population dynamics respond to different environmental processes: colonies in regions with lower riverine discharge are primarily affected by drought, while floods have a greater impact on those in areas with higher discharge. However, seabirds in coastal regions with minimal discharge can still face flooding risks from runoff, increased turbidity, and altered foraging ranges affecting breeding success (de la Cruz *et al*., 2021, 2022). Likewise, even areas with high freshwater input can be susceptible to drought effects (Anderson *et al*., 2021). Sustained climate pressures on river systems typically have a more significant impact on seabird population size than do short‐term events (Clark‐Wolf *et al*., 2023). Therefore, adopting a framework that assesses regions based on freshwater input magnitude, such as the 1000 m^3^ s^−1^ threshold proposed by Jutla *et al*. (2011) alongside regional hydrological trends, could improve research and conservation efforts for seabirds affected by different river types (Milliman *et al*., 2008).

### (5) River effects during extrinsic disturbances

River–seabird relationships can be affected by a range of extrinsic disturbances (climatic and/or anthropogenic), which can either enhance or disrupt the ecological role of rivers for seabirds. In some contexts, rivers can act as stabilising features. For example, during marine heatwaves or resource‐poor conditions, river plumes can represent thermal refuges or provide a more predictable source of prey (Loredo *et al*., 2019; Seher *et al*., 2022). For little penguins, drought impacts varied by colony depending on the adjacent river, with less‐regulated rivers linked to more stable reproductive outcomes (Kowalczyk *et al*., 2015b; Bice *et al*., 2017; Colombelli‐Négrel *et al*., 2022). Similarly, studies on Magellanic penguins suggest that naturally reduced river flow can support breeding and buffer short‐term impacts of ocean warming and extreme events like marine heatwaves and storms (Rebstock & Boersma, 2018; Clark‐Wolf *et al*., 2023).

While studies in temperate regions mostly reported positive effects of rivers during floods and storms (Waggitt *et al*., 2020; Clark‐Wolf *et al*., 2023), negative impacts, such as increased seabird mortality during extreme weather events, were found for tropical regions (Tavares *et al*., 2016). Nesting near a river mouth can expose seabirds to flooding risks (Whittington *et al*., 2016), with elevation potentially influencing breeding success (Bried & Jouventin, 2001). Nevertheless, shifts towards nesting in river‐influenced areas were not uncommon (Newson *et al*., 2013), and freshwater plumes often became more important during anomalous rainfall and floods (Ribic *et al*., 1997). Observations of new colonies near river mouths, increased reports of inland vagrancy and estuarine ornithogenic inputs suggest that river systems may offer critical resources as marine environments degrade (Häkkinen *et al*., 2021; Signa, Mazzola & Vizzini, 2021; Baumann *et al*., 2023). Estuaries and river mouths already serve as important breeding and foraging habitats for many charadriiform species (Ribic *et al*., 1997; Lopes, 2014; Suzuki *et al*., 2019), with hypothesised superabundant prey availability supporting both the formation of a new colony between productive estuaries (Dunlop & McNeill, 2017) and the sharing of resources between sympatric congeners (Peck‐Richardson *et al*., 2018). If seabird recruitment increases near plumes, it may signal that rivers are becoming key refuges under climate change (Cama *et al*., 2012; Seher *et al*., 2022), although shifts toward riverine food webs may also reflect anthropogenic pressures such as marine overfishing (Elliott & Elliott, 2016). Overall, rivers are implicated less often in climate change disturbances than in mitigation of them, with compounding impacts appearing more severe in tropical regions than temperate ones. Given the absence of global trends in hydrological cycle changes, regional climate and weather extremes must be considered when evaluating interactions between rivers and seabirds (Milliman *et al*., 2008).

Human‐altered river systems add further complexity. For instance, contaminant‐laden discharges such as those following dam failures can introduce pollutants into seabird prey across large spatial scales, even where seabirds forage offshore, causing long‐term impacts whose full extent remains unknown (Nunes *et al*., 2022; de Barros Bauer *et al*., 2024). Management actions, such as relocating colonies within estuarine systems, have also shown mixed outcomes, with river flow patterns influencing foraging effort and diet (Anderson *et al*., 2007; Lyons, 2010). Conversely, efforts to restore rivers and improve fish stocks have the potential to benefit both freshwater and marine predators, although assumptions about positive seabird outcomes require more robust evidence (Dias, Frisk & Jordaan, 2019a, 2022).

With more than 40% of global river flows intercepted by dams (Wu *et al*., 2023), and with climate change expected to increase the frequency and intensity of extreme events (Deininger & Frigstad, 2019), the role of rivers as ecological connectors between land and sea will likely grow in significance. Species that can exploit these dynamic systems may gain a relative advantage, while others may face new challenges. Rivers are increasingly recognised not just as stressors but also as critical support zones for seabirds facing mounting environmental pressures. These patterns suggest that while rivers can buffer seabirds against short‐term environmental variability, they may also introduce risks depending on regional hydrology and disturbance regimes. Exploring how seabirds respond to riverine features could yield valuable insights into how marine predators adjust their foraging strategies and habitat use in an increasingly dynamic seascape.

### (6) Future research and conservation/management actions

Given the global decline of seabird populations and the uneven impacts of climate change on riverine and coastal systems (Croxall *et al*., 2012; Ramírez *et al*., 2017; Dias *et al*., 2019b), combined with the potential for anthropogenic actions to intensify these pressures, safeguarding seabird species requires collaborative, multidisciplinary action. This includes integrating land and marine management, water resources, fisheries, and conservation science (Couto & Sethi, 2024). Optimising protected area networks by reassessing and augmenting zones that harbour key seabird habitats, such as estuaries and river plumes, will be critical to support vulnerable populations as climate change and human activities increasingly alter river flows and coastal foraging conditions (Ramos & Pereira, 2022; Hansen *et al*., 2023).

Recent findings reinforce that while rivers can increase pollutant exposure through trophic pathways, they may also become critical foraging refuges as marine conditions deteriorate. Thus, riverine ecosystems could play a growing role in seabird resilience, depending on local watershed characteristics and management.

Regular monitoring of seabird responses to river runoff, climate variability, and human pressures will provide invaluable data for adaptive management strategies.

To address data limitations and research gaps, we suggest multiple possible approaches: (*i*) establishing river flow‐gauge stations globally to generate discharge time series for hydrodynamic modelling and assessment of river‐to‐sea dynamics (Teng *et al*., 2017); (*ii*) conducting targeted studies on turbidity effects across different flow periods to understand their influences on foraging and breeding (Braby, Underhill & Simmons, 2011; Collar *et al*., 2017; Phillips *et al*., 2021; Wells *et al*., 2017); (*iii*) expanding demographic and health studies, including physiological and contaminant assessments, to identify species‐specific thresholds and clarify the role of rivers as vectors of pollutants and pathogens (MacDonald, 2006; Meijer *et al*., 2021; Campbell *et al*., 2022); (*iv*) investigating how river flows interact with climate change, using long‐term data sets of single species across regions (Häkkinen *et al*., 2021; Signa *et al*., 2021); (*v*) evaluating effects of current river management actions on seabirds, including both positive and negative outcomes (Ibáñez, Caiola & Belmar, 2020; Wu *et al*., 2023); and (*vi*) expanding geographic and taxonomic scope, particularly focusing on research in Africa, Asia, and tropical regions, where hydrological, oceanographic, and anthropogenic threats are emerging, and where research gaps reflect broader inequities in funding and capacity.

To enable cross‐study comparisons and future meta‐analyses, we encourage researchers to adopt shared definitions and consistently report core measurements. Where feasible, studies should: (*i*) collect coordinated measurements of river outflow and plume‐related oceanographic variables (e.g. SST, SSS, Chl‐a, turbidity) across multiple temporal scales to clarify the numerous pathways by which rivers influence seabirds; (*ii*) report the long‐term and contemporary mean monthly outflow (m^3^ s^−1^) of all rivers considered relevant to the study area and focal taxa, whether or not outflow is directly analysed as a predictor variable. Providing this hydrological context, even when outflow is not the primary variable, supports cross‐study comparability and more robust ecological interpretation. For example, when using ‘number of rivers’, weighted measures that incorporate river size/discharge are preferable to simple counts. Likewise, when applying distance metrics, clearly specify the size or class of the rivers to which distances refer, as the ecological significance of distance varies with river magnitude and foraging guild. Evaluating distances to rivers across multiple size classes may reduce the risk of overlooking relationships due to scale mismatches in variable definitions. (*iii*) Conduct ocean‐based prey abundance surveys in both river‐influenced and control marine areas to elucidate trophic pathways. (*iv*) Include stable isotope analyses of predator and prey tissues incorporating δ^34^S alongside δ^13^C and δ^15^N in diet studies, establishing and reporting local baselines. (*v*) Establish demographic time‐series data sets linking seabird data to river discharge and large‐scale climate indices (e.g. ENSO, PDO) through consistent long‐term monitoring (≥20 years) to support robust trend detection.

Although these recommendations fall short of full standardisation, they provide a framework for improving consistency and transparency. Table 2 offers examples of metrics that have been most informative in previous studies. Adopting these practices can help lay the groundwork for comparative analyses and more effective conservation strategies as river management and climate change reshape coastal ecosystems.

Finally, mitigating human impacts remains essential, by reducing pollution, managing water extraction, restoring riverbanks, improving water quality, and regulating fisheries. Context‐specific strategies are key, as rivers can act as both sources of risk and resilience for seabirds depending on local conditions. These efforts will be critical to sustain healthy ecosystems supporting seabirds facing accelerating pressures (Couto & Sethi, 2024).

## V. CONCLUSIONS

(1) Evidence of river effects spans multiple seabird research priority areas, seabird orders, and climate regions, underscoring the broad ecological relevance of freshwater inputs to seabird ecology. However, the limited use of river‐specific metrics in comparison to oceanographic variables reveals a persistent gap in how freshwater influence is quantified at sea. Integrating riverine parameters more routinely will improve understanding and predictive power across ecological scales.
(2) Incorporating both terrestrial/freshwater and marine environmental variables into seabird foraging studies, within multi‐variable study frameworks, is critical to capturing the full range of ecological drivers. Establishing shared definitions and consistently reporting river metrics such as outflow, plume extent, and river proximity will enhance cross‐study comparability and enable robust meta‐analyses of river effects.
(3) River plumes represent key foraging habitats for seabirds, enhancing prey accessibility and promoting site fidelity in otherwise variable environments. Their role in driving foraging intensity and supporting new colony formation highlights the importance of protecting estuarine and river‐influenced zones under shifting climate and ocean conditions.
(4) Diet studies consistently show increased prey diversity in estuarine waters relative to marine areas. Incorporating isotopic tools, particularly δ^34^S, can strengthen the use of seabird diet as a proxy for forage fish dynamics and river influence, offering a valuable sentinel function across freshwater–marine gradients.
(5) Riverine areas can pose significant ecological trade‐offs, supporting foraging yet increasing exposure to pollutants and fisheries interactions. Although concern about pollution is growing, studies directly linking riverine exposure to seabird health outcomes remain scarce and should be prioritised.
(6) Demographic effects of rivers on seabirds appear contingent on river magnitude and environmental thresholds (e.g. turbidity, outflow), which shape foraging opportunities and breeding success. Long‐term demographic monitoring, coupled with regional hydrological data and climate indices, is critical to detect population‐level responses and inform adaptive management.
(7) As climate change and human pressures intensify, river‐influenced habitats may increasingly function as climate refuges. This is reflected in growing research interest, reports of colony redistribution, and the apparent role of plumes in buffering environmental variability. Monitoring changes in seabird colonies, whether in size, location, diet composition, or health, can help confirm emerging shifts in habitat use and foraging ecology driven by coastal food web alterations in river‐influenced systems.
(8) Future research should expand geographic and taxonomic representation, especially in data‐poor tropical systems facing compounding hydrological and climate risks. Investigating how rivers modulate seabird resilience in these regions will be key to shaping inclusive and forward‐looking conservation strategies.

## Supporting information


**Table S1.** Results of the systematic review and rationale for the selected studies (*N* = 166).
**Table S2.** Information extracted from the selected papers, including environmental variables related to seabird biological responses (*N* = 51).
**Table S3.** Evidence of river effects on seabirds across 51 papers included in the review, with research findings summarised to support evidence type classification (70 river effect entries).
**Table S4.** Single‐species study records (62 species; 115 total observations), one entry per species per paper, with associated study characteristics.
**Table S5.** Relationship direction between river outflow (or plume strength) and seabird demographics across rivers of different magnitudes (based on mean monthly river outflow, m^3^ s^−1^) (*N* = 10 studies).

## Data Availability

The data that supports the findings of this study are available in the supplementary material of this article.

## References

[brv70143-bib-0001] Afán, I. , Arcos, J. M. , Ramírez, F. , García, D. , Rodríguez, B. , Delord, K. , Boué, A. , Micol, T. , Weimerskirch, H. & Louzao, M. (2021). Where to head: environmental conditions shape foraging destinations in a critically endangered seabird. Marine Biology 168, 1–10.

[brv70143-bib-0002] Ainley, D. G. & Wilson, R. P. (2023). The Aquatic World of Penguins: Biology of Fish‐Birds. Springer, Cham.

[brv70143-bib-0003] Ainley, D. G. , Ribic, C. A. & Woehler, E. J. (2012). Adding the ocean to the study of seabirds: a brief history of at‐sea seabird research. Marine Ecology Progress Series 451, 231–243.

[brv70143-bib-0004] Anderson, A. M. , Friis, C. , Gratto‐Trevor, C. L. , Harris, C. M. , Love, O. P. , Morrison, R. G. , Prosser, S. W. , Nol, E. & Smith, P. A. (2021). Drought at a coastal wetland affects refuelling and migration strategies of shorebirds. Oecologia 197, 661–674. 34657196 10.1007/s00442-021-05047-x PMC8585834

[brv70143-bib-0005] Anderson, S. K. , Roby, D. D. , Lyons, D. E. & Collis, K. (2007). Relationship of Caspian tern foraging ecology to nesting success in the Columbia River estuary, Oregon, USA. Estuarine, Coastal and Shelf Science 73, 447–456.

[brv70143-bib-0006] Armstrong, J. B. , Schindler, D. E. , Cunningham, C. J. , Deacy, W. & Walsh, P. (2020). Watershed complexity increases the capacity for salmon–wildlife interactions in coastal ecosystems. Conservation Letters 13, e12689.

[brv70143-bib-0007] Assali, C. , Bez, N. & Tremblay, Y. (2020). Raking the ocean surface: new patterns of coordinated motion in seabirds. Journal of Avian Biology 51, e02258.

[brv70143-bib-0008] Atwood, E. C. , Falcieri, F. M. , Piehl, S. , Bochow, M. , Matthies, M. , Franke, J. , Carniel, S. , Sclavo, M. , Laforsch, C. & Siegert, F. (2019). Coastal accumulation of microplastic particles emitted from the Po River, Northern Italy: comparing remote sensing and hydrodynamic modelling with in situ sample collections. Marine Pollution Bulletin 138, 561–574. 30660307 10.1016/j.marpolbul.2018.11.045

[brv70143-bib-0009] Auricht, H. , Mosley, L. , Lewis, M. & Clarke, K. (2022). Mapping the long‐term influence of river discharge on coastal ocean chlorophyll‐a. Remote Sensing in Ecology and Conservation 8, 629–643.

[brv70143-bib-0010] Ballance, L. , Ainley, D. G. & Hunt, G. L. Jr. (2001). Seabird foraging ecology. In Encyclopedia of Ocean Sciences (Volume 5), pp. 2636–2644. Elsevier, Amsterdam.

[brv70143-bib-0011] Barrett, J. (2008). The influence of oceanographic and terrestrial attributes on marbled murrelet (*Brachyramphus marmoratus*) marine habitat selection during the breeding season. Master's thesis, Simon Fraser University, Burnaby.

[brv70143-bib-0012] Baumann, M. J. , Brant, S. V. , Bauernfeind, S. M. , Gerhart, C. R. , Williamson, J. L. , Johnson, A. B. , Spellman, G. M. , Uhrig, S. R. , West, S. & Witt, C. C. (2023). Freshwater parasites as potential barriers to seabird dispersal: evidence from vagrant booby specimens in western North America. The Wilson Journal of Ornithology 135, 327–344.

[brv70143-bib-0013] Bearhop, S. , Thompson, D. R. , Waldron, S. , Russell, I. C. , Alexander, G. & Furness, R. W. (1999). Stable isotopes indicate the extent of freshwater feeding by cormorants *Phalacrocorax carbo* shot at inland fisheries in England. Journal of Applied Ecology 36, 75–84.

[brv70143-bib-0014] Beavis, S. G. , Wong, V. N. , Mosley, L. M. , Baldwin, D. S. , Latimer, J. O. , Lane, P. & Lal, A. (2023). Water quality risks in the Murray‐Darling basin. Australasian Journal of Water Resources 27, 85–102.

[brv70143-bib-0015] Belkin, I. M. & Cornillon, P. C. (2007). Fronts in the world ocean's large marine ecosystems. ICES CM 500, 21.

[brv70143-bib-0016] Benemann, V. R. F. , Ribeiro, B. C. , Moreira, E. G. & Petry, M. V. (2024). Differences in mercury (THg) levels in brown booby (*Sula leucogaster*) feathers from two environmentally distinct Brazilian archipelagos. Science of the Total Environment 954, 176457. 39343391 10.1016/j.scitotenv.2024.176457

[brv70143-bib-0017] Bice, C. M. , Gibbs, M. S. , Kilsby, N. N. , Mallen‐Cooper, M. & Zampatti, B. P. (2017). Putting the ‘river’ back into the lower river Murray: quantifying the hydraulic impact of river regulation to guide ecological restoration. Transactions of the Royal Society of South Australia 141, 108–131.

[brv70143-bib-0018] Binkowski, L. J. , Fort, J. , Brault‐Favrou, M. , Gallien, F. , Le Guillou, G. , Chastel, O. & Bustamante, P. (2021). Foraging ecology drives mercury contamination in chick gulls from the English Channel. Chemosphere 267, 128622. 33162157 10.1016/j.chemosphere.2020.128622

[brv70143-bib-0019] Boersma, P. D. (2008). Penguins as marine sentinels. Bioscience 58, 597–607.

[brv70143-bib-0020] Boutin, J. , Martin, N. , Reverdin, G. , Yin, X. & Gaillard, F. (2013). Sea surface freshening inferred from SMOS and ARGO salinity: impact of rain. Ocean Science 9, 183–192.

[brv70143-bib-0021] Braby, J. , Underhill, L. & Simmons, R. (2011). Prey capture success and chick diet of Damara terns *Sterna balaenarum* in Namibia. African Journal of Marine Science 33, 247–254.

[brv70143-bib-0022] Brasso, R. L. , Chiaradia, A. , Polito, M. J. , Rey, A. R. & Emslie, S. D. (2015). A comprehensive assessment of mercury exposure in penguin populations throughout the southern hemisphere: using trophic calculations to identify sources of population‐level variation. Marine Pollution Bulletin 97, 408–418. 26072048 10.1016/j.marpolbul.2015.05.059

[brv70143-bib-0023] Bried, J. & Jouventin, P. (2001). The king penguin *Aptenodytes patagonicus*, a non‐nesting bird which selects its breeding habitat. Ibis 143, 670–673.

[brv70143-bib-0024] Brisson‐Curadeau, É. & Elliott, K. H. (2019). Prey capture and selection throughout the breeding season in a deep‐diving generalist seabird, the thick‐billed murre. Journal of Avian Biology 50, e01930.

[brv70143-bib-0025] Broadley, A. , Stewart‐Koster, B. , Burford, M. A. & Brown, C. J. (2022). A global review of the critical link between river flows and productivity in marine fisheries. Reviews in Fish Biology and Fisheries 32, 805–825.

[brv70143-bib-0026] Burger, J. (2023). Estuarine and coastal birds, climate change, and sea‐level rise. In Climate Change and Estuaries. CRC Press, Boca Raton.

[brv70143-bib-0027] Burrows, M. T. , Schoeman, D. S. , Buckley, L. B. , Moore, P. , Poloczanska, E. S. , Brander, K. M. , Brown, C. , Bruno, J. F. , Duarte, C. M. & Halpern, B. S. (2011). The pace of shifting climate in marine and terrestrial ecosystems. Science 334, 652–655. 22053045 10.1126/science.1210288

[brv70143-bib-0028] Cama, A. , Abellana, R. , Christel, I. , Ferrer, X. & Vieites, D. R. (2012). Moving to the sea: a challenge for an inshore species, the slender‐billed gull. Marine Ecology Progress Series 463, 285–295.

[brv70143-bib-0029] Campbell, K. , Paparini, A. , Gomez, A. B. , Cannell, B. & Stephens, N. (2022). Fatal toxoplasmosis in little penguins (*Eudyptula minor*) from Penguin Island, Western Australia. International Journal for Parasitology: Parasites and Wildlife 17, 211–217. 35198375 10.1016/j.ijppaw.2022.02.006 PMC8850582

[brv70143-bib-0030] Carr, M. H. , Neigel, J. E. , Estes, J. A. , Andelman, S. , Warner, R. R. & Largier, J. L. (2003). Comparing marine and terrestrial ecosystems: implications for the design of coastal marine reserves. Ecological Applications 13, 90–107.

[brv70143-bib-0031] Champoux, L. & Boily, M. (2017). Temporal trends of mercury and organohalogen contaminants in great blue heron eggs from the St. Lawrence River, Québec, Canada, 1991–2011, and relationships with tracers of feeding ecology. Science of the Total Environment 609, 1270–1285. 28797142 10.1016/j.scitotenv.2017.07.223

[brv70143-bib-0032] Clark‐Wolf, T. , Dee Boersma, P. , Rebstock, G. A. & Abrahms, B. (2023). Climate presses and pulses mediate the decline of a migratory predator. Proceedings of the National Academy of Sciences 120, e2209821120. 10.1073/pnas.2209821120 PMC9934075 36623194

[brv70143-bib-0033] Clatterbuck, C. A. , Lewison, R. L. , Orben, R. A. , Ackerman, J. T. , Torres, L. G. , Suryan, R. M. , Warzybok, P. , Jahncke, J. & Shaffer, S. A. (2021). Foraging in marine habitats increases mercury concentrations in a generalist seabird. Chemosphere 279, 130470. 34134398 10.1016/j.chemosphere.2021.130470

[brv70143-bib-0034] Cloern, J. E. , Jassby, A. D. , Schraga, T. S. , Nejad, E. & Martin, C. (2017). Ecosystem variability along the estuarine salinity gradient: examples from long‐term study of San Francisco Bay. Limnology and Oceanography 62, S272–S291.

[brv70143-bib-0035] Collar, S. , Roby, D. D. & Lyons, D. E. (2017). Top‐down and bottom‐up interactions influence fledging success at North America's largest colony of Caspian terns (*Hydroprogne caspia*). Estuaries and Coasts 40, 1808–1818.

[brv70143-bib-0036] Colombano, D. D. , Carlson, S. M. , Hobbs, J. A. & Ruhi, A. (2022). Four decades of climatic fluctuations and fish recruitment stability across a marine‐freshwater gradient. Global Change Biology 28, 5104–5120. 35583053 10.1111/gcb.16266 PMC9545339

[brv70143-bib-0037] Colombelli‐Négrel, D. , Nur, D. , Auricht, H. C. , Clarke, K. D. , Mosley, L. M. & Dann, P. (2022). Combined effects of hydrological drought and reduced food availability on the decline of the little penguins in South Australia. Frontiers in Marine Science 9, 875259.

[brv70143-bib-0038] Costa, P. L. , Bugoni, L. , Kinas, P. G. & Madureira, L. A. S. P. (2020). Seabirds, environmental features and the Argentine anchovy *Engraulis anchoita* in the southwestern Atlantic Ocean. Marine Ecology Progress Series 651, 199–213.

[brv70143-bib-0039] Coulson, J. C. (2016). A review of philopatry in seabirds and comparisons with other waterbird species. Waterbirds 39, 229–240.

[brv70143-bib-0040] Couto, T. B. & Sethi, S. A. (2024). River‐to‐sea ecosystem management. Nature Sustainability 7, 1–3.

[brv70143-bib-0041] Cox, S. , Embling, C. , Hosegood, P. , Votier, S. & Ingram, S. (2018). Oceanographic drivers of marine mammal and seabird habitat use across shelf seas: a guide to key features and recommendations for future research and conservation management. Estuarine, Coastal and Shelf Science 212, 294–310.

[brv70143-bib-0042] Croxall, J. P. , Butchart, S. H. , Lascelles, B. , Stattersfield, A. J. , Sullivan, B. , Symes, A. & Taylor, P. (2012). Seabird conservation status, threats and priority actions: a global assessment. Bird Conservation International 22, 1–34.

[brv70143-bib-0043] Cumming, G. S. , Paxton, M. , King, J. & Beuster, H. (2012). Foraging guild membership explains variation in waterbird responses to the hydrological regime of an arid‐region flood‐pulse river in Namibia. Freshwater Biology 57, 1202–1213.

[brv70143-bib-0044] d'Alcalà, M. R. (2019). Similarities, differences and mechanisms of climate impact on terrestrial vs. marine ecosystems. Nature Conservation 34, 505–523.

[brv70143-bib-0045] da Costa, Á. K. R. , Pereira, L. C. C. , Jiménez, J. A. , de Oliveira, A. R. G. , Jesus Flores‐Montes, M. & da Costa, R. M. (2022). Effects of extreme climatic events on the hydrological parameters of the estuarine waters of the Amazon coast. Estuaries and Coasts 45, 1517–1533.

[brv70143-bib-0046] Darby, J. , Clairbaux, M. , Bennison, A. , Quinn, J. & Jessopp, M. (2022). Underwater visibility constrains the foraging behaviour of a diving pelagic seabird. Proceedings of the Royal Society B: Biological Sciences 289, 20220862. 10.1098/rspb.2022.0862 PMC9277241 35858070

[brv70143-bib-0047] Daudt, N. W. , Martins, S. P. , Kirinus, E. P. & Bugoni, L. (2019). Seabird assemblage at the mouth of the Amazon River and its relationship with environmental characteristics. Journal of Sea Research 155, 101826.

[brv70143-bib-0048] Day, R. D. , Roseneau, D. G. , Vander Pol, S. S. , Hobson, K. A. , Donard, O. F. , Pugh, R. S. , Moors, A. J. & Becker, P. R. (2012). Regional, temporal, and species patterns of mercury in Alaskan seabird eggs: mercury sources and cycling or food web effects? Environmental Pollution 166, 226–232. 22522226 10.1016/j.envpol.2012.03.004

[brv70143-bib-0049] de Barros Bauer, A. , Andra Linhares, B. , Nunes, G. T. , Costa, P. G. , Zebral, Y. D. , Bianchini, A. & Bugoni, L. (2024). Temporal changes in metal and arsenic concentrations in blood and feathers of tropical seabirds after one of the largest environmental disasters associated with mining. Environmental Research 248, 118240. 38266903 10.1016/j.envres.2024.118240

[brv70143-bib-0050] de la Cruz, A. , Ramos, F. , Navarro, G. , Cózar, A. , Bécares, J. & Arroyo, G. M. (2021). Drivers for spatial modelling of a critically endangered seabird on a dynamic ocean area: Balearic shearwaters are non‐vegetarian. Aquatic Conservation: Marine and Freshwater Ecosystems 31, 1700–1714.

[brv70143-bib-0051] de la Cruz, A. , Ramos, F. , Tornero, J. , Rincón, M. M. , Jiménez, M. P. & Arroyo, G. M. (2022). Seabird distribution is better predicted by abundance of prey than oceanography: a case study in the Gulf of Cadiz (SW Iberian Peninsula). ICES Journal of Marine Science 79, 204–217.

[brv70143-bib-0052] Deininger, A. & Frigstad, H. (2019). Reevaluating the role of organic matter sources for coastal eutrophication, oligotrophication, and ecosystem health. Frontiers in Marine Science 6, 210.

[brv70143-bib-0053] Dias, B. S. , Frisk, M. G. & Jordaan, A. (2019 *a*). Opening the tap: increased riverine connectivity strengthens marine food web pathways. PLoS One 14, e0217008. 31120934 10.1371/journal.pone.0217008 PMC6532889

[brv70143-bib-0054] Dias, B. S. , Frisk, M. G. & Jordaan, A. (2022). Contrasting fishing effort reduction and habitat connectivity as management strategies to promote alewife (*Alosa pseudoharengus*) recovery using an ecosystem model. Limnology and Oceanography 67, S5–S22.

[brv70143-bib-0055] Dias, E. , Morais, P. , Leopold, M. , Campos, J. & Antunes, C. (2012). Natural born indicators: great cormorant *Phalacrocorax carbo* (Aves: Phalacrocoracidae) as monitors of river discharge influence on estuarine ichthyofauna. Journal of Sea Research 73, 101–108.

[brv70143-bib-0056] Dias, M. P. , Martin, R. , Pearmain, E. J. , Burfield, I. J. , Small, C. , Phillips, R. A. , Yates, O. , Lascelles, B. , Borboroglu, P. G. & Croxall, J. P. (2019 *b*). Threats to seabirds: a global assessment. Biological Conservation 237, 525–537.

[brv70143-bib-0057] Diaz‐Santibañez, I. , Clark, B. L. & Zavalaga, C. B. (2023). Guanay cormorant (*Leucocarbo bougainvilliorum*) pellets as an indicator of marine plastic pollution along the Peruvian coast. Marine Pollution Bulletin 192, 115104. 37301006 10.1016/j.marpolbul.2023.115104

[brv70143-bib-0058] Drinkwater, K. F. & Frank, K. T. (1994). Effects of river regulation and diversion on marine fish and invertebrates. Aquatic Conservation: Marine and Freshwater Ecosystems 4, 135–151.

[brv70143-bib-0059] Dunlop, J. N. & McNeill, S. (2017). Local movements, foraging patterns, and heavy metals exposure in Caspian terns *Hydroprogne caspia* breeding on Penguin Island, Western Australia. Marine Ornithology 45, 115–120.

[brv70143-bib-0060] Elliott, J. E. , Noble, D. G. , Norstrom, R. & Whitehead, P. (1989). Organochlorine contaminants in seabird eggs from the Pacific coast of Canada, 1971–1986. Environmental Monitoring and Assessment 12, 67–82. 24249067 10.1007/BF00396737

[brv70143-bib-0061] Elliott, K. H. & Elliott, J. E. (2016). Origin of sulfur in diet drives spatial and temporal mercury trends in seabird eggs from Pacific Canada 1968–2015. Environmental Science & Technology 50, 13380–13386. 27993060 10.1021/acs.est.6b05458

[brv70143-bib-0062] Emelyanov, E. M. (2005). The Barrier Zones in the Ocean. Springer Science & Business Media, Berlin.

[brv70143-bib-0063] Evans, R. , Lea, M. A. & Hindell, M. A. (2021). Predicting the distribution of foraging seabirds during a period of heightened environmental variability. Ecological Applications 31, e02343. 33817895 10.1002/eap.2343

[brv70143-bib-0064] Fan, J. , Yang, J. , He, Y. & Jiang, X. (2023). Diel, seasonal, and annual variations of fish assemblages in intertidal creeks of the Changjiang River estuary. Journal of Oceanology and Limnology 41, 1–15.

[brv70143-bib-0065] Farinós‐Celdrán, P. , Robledano‐Aymerich, F. & Palazón‐Ferrando, J. (2019). Stable isotope analysis reveals the feeding distribution of wintering great cormorant *Phalacrocorax carbo sinensis* along a marine‐continental Mediterranean gradient. Estuarine, Coastal and Shelf Science 216, 157–164.

[brv70143-bib-0066] Fasola, M. , Bogliani, G. , Saino, N. & Canova, L. (1989). Foraging, feeding and time‐activity niches of eight species of breeding seabirds in the coastal wetlands of the Adriatic Sea. Italian Journal of Zoology 56, 61–72.

[brv70143-bib-0067] Finger, A. , Lavers, J. L. , Orbell, J. D. , Dann, P. , Nugegoda, D. & Scarpaci, C. (2016). Seasonal variation and annual trends of metals and metalloids in the blood of the little penguin (*Eudyptula minor*). Marine Pollution Bulletin 110, 261–273. 27329818 10.1016/j.marpolbul.2016.06.055

[brv70143-bib-0068] Foden, W. B. , Young, B. E. , Akçakaya, H. R. , Garcia, R. A. , Hoffmann, A. A. , Stein, B. A. , Thomas, C. D. , Wheatley, C. J. , Bickford, D. & Carr, J. A. (2019). Climate change vulnerability assessment of species. Wiley Interdisciplinary Reviews: Climate Change 10, e551.

[brv70143-bib-0069] Franks, D. W. , Ruxton, G. D. & Sherratt, T. (2025). Ecology needs a causal overhaul. Biological Reviews 100, e70029. 10.1111/brv.70029 40344451

[brv70143-bib-0070] Fredston‐Hermann, A. , Brown, C. J. , Albert, S. , Klein, C. J. , Mangubhai, S. , Nelson, J. L. , Teneva, L. , Wenger, A. , Gaines, S. D. & Halpern, B. S. (2016). Where does river runoff matter for coastal marine conservation? Frontiers in Marine Science 3, 273.

[brv70143-bib-0071] Garcia‐Heras, M.‐S. , Wolf, C. , Guerrero, J. A. B. , Adrean, L. J. , Nelson, S. K. , Roby, D. D. , Betts, M. G. & Rivers, J. W. (2024). Marine habitat use and movement in response to ocean warming by a threatened forest‐nesting seabird. Global Ecology and Conservation 47, e02857.

[brv70143-bib-0072] Garthe, S. (1997). Influence of hydrography, fishing activity, and colony location on summer seabird distribution in the south‐eastern North Sea. ICES Journal of Marine Science 54, 566–577.

[brv70143-bib-0073] Gillanders, B. M. & Kingsford, M. J. (2002). Impact of changes in flow of freshwater on estuarine and open coastal habitats and the associated organisms. Oceanography and Marine Biology: An Annual Review 40, 233–283.

[brv70143-bib-0074] Gladics, A. J. , Suryan, R. M. , Parrish, J. K. , Horton, C. A. , Daly, E. A. & Peterson, W. T. (2015). Environmental drivers and reproductive consequences of variation in the diet of a marine predator. Journal of Marine Systems 146, 72–81.

[brv70143-bib-0075] Golet, G. H. , Kuletz, K. J. , Roby, D. D. & Irons, D. B. (2000). Adult prey choice affects chick growth and reproductive success in pigeon guillemots. Auk 117, 82–91.

[brv70143-bib-0076] González Carman, V. , Mandiola, A. , Alemany, D. , Dassis, M. , Seco Pon, J. P. , Prosdocimi, L. , Ponce De León, A. , Mianzan, H. , Acha, E. M. & Rodríguez, D. (2016). Distribution of megafaunal species in the southwestern Atlantic: key ecological areas and opportunities for marine conservation. ICES Journal of Marine Science 73, 1579–1588.

[brv70143-bib-0077] Grant, M. L. , Bond, A. L. & Lavers, J. L. (2022). The influence of seabirds on their breeding, roosting and nesting grounds: a systematic review and meta‐analysis. Journal of Animal Ecology 91, 1–23. 10.1111/1365-2656.13699 PMC9324971 35395097

[brv70143-bib-0078] Greenwell, C. , Tweedley, J. , Moore, G. , Lenanton, R. , Dunlop, J. & Loneragan, N. (2021). Feeding ecology of a threatened coastal seabird across an inner shelf seascape. Estuarine, Coastal and Shelf Science 263, 107627.

[brv70143-bib-0079] Gupta, H. , Reddy, S. K. K. & Gandla, V. K. (2023). Temporal trends in water discharge characteristics of the large peninsular rivers: assessing the role of climatic and anthropogenic factors. In Climate Change and Environmental Impacts: Past, Present and Future Perspective, pp. 321–331. Springer, Cham.

[brv70143-bib-0080] Häkkinen, H. , Petrovan, S. O. , Sutherland, W. J. & Pettorelli, N. (2021). Terrestrial or marine species distribution model: why not both? A case study with seabirds. Ecology and Evolution 11, 16634–16646. 34938462 10.1002/ece3.8272 PMC8668722

[brv70143-bib-0081] Haney, J. C. & Stone, A. E. (1988). Seabird foraging tactics and water clarity: are plunge divers really in the clear? Marine Ecology Progress Series 49, 1–9.

[brv70143-bib-0082] Hansen, H. H. , Bergman, E. , Cowx, I. G. , Lind, L. , Pauna, V. H. & Willis, K. A. (2023). Resilient rivers and connected marine systems: a review of mutual sustainability opportunities. Global Sustainability 6, 1–71.37692862

[brv70143-bib-0083] Harari, M. B. , Parola, H. R. , Hartwell, C. J. & Riegelman, A. (2020). Literature searches in systematic reviews and meta‐analyses: a review, evaluation, and recommendations. Journal of Vocational Behavior 118, 103377.

[brv70143-bib-0084] Haynes, T. B. , Nelson, S. K. , Poulsen, F. & Padula, V. (2011). Spatial distribution and habitat use of marbled Murrelets *Brachyramphus marmoratus* at sea in port Snettisham, Alaska. Marine Ornithology 39, 151–162.

[brv70143-bib-0085] Henkel, L. A. (2006). Effect of water clarity on the distribution of marine birds in nearshore waters of Monterey Bay, California. Journal of Field Ornithology 77, 151–156.

[brv70143-bib-0086] Hernández, N. , Oro, D. & Sanz‐Aguilar, A. (2017). Environmental conditions, age, and senescence differentially influence survival and reproduction in the storm petrel. Journal of Ornithology 158, 113–123.

[brv70143-bib-0087] Higgins, J. V. , Touval, J. L. , Unnasch, R. S. , Reichle, S. , Oren, D. C. , Hoekstra, J. M. & Cleary, D. (2006). Who needs to spend money on conservation science anyway? Conservation Biology 20, 1566–1568. 17181787 10.1111/j.1523-1739.2006.00583_1.x

[brv70143-bib-0088] Hostetter, N. J. , Evans, A. F. , Payton, Q. , Roby, D. D. , Lyons, D. E. & Collis, K. (2023). A review of factors affecting the susceptibility of juvenile salmonids to avian predation. North American Journal of Fisheries Management 43, 244–256.

[brv70143-bib-0089] Hughes, B. B. , Beas‐Luna, R. , Barner, A. K. , Brewitt, K. , Brumbaugh, D. R. , Cerny‐Chipman, E. B. , Close, S. L. , Coblentz, K. E. , De Nesnera, K. L. & Drobnitch, S. T. (2017). Long‐term studies contribute disproportionately to ecology and policy. Bioscience 67, 271–281.

[brv70143-bib-0090] Hunt, G. L. , Mehlum, F. , Russell, R. W. , Irons, D. , Decker, B. & Becker, P. H. (1999). Physical processes, prey abundance, and the foraging ecology of seabirds. In Proceedings of the 22nd International Ornithological Congress, p. S34–3. BirdLife South Africa, Durban.

[brv70143-bib-0091] Ibáñez, C. , Caiola, N. & Belmar, O. (2020). Environmental flows in the lower Ebro River and Delta: current status and guidelines for a holistic approach. Water 12, 2670.

[brv70143-bib-0092] International Union For Conservation Of Nature (IUCN) (2025). *The IUCN Red List of Threatened Species* (Version 2025–1). https://www.iucnredlist.org [accessed 28 October 2025].

[brv70143-bib-0093] Jodice, P. G. & Suryan, R. M. (2010). The transboundary nature of seabird ecology. In Landscape‐Scale Conservation Planning, pp. 139–165. Springer, Berlin.

[brv70143-bib-0094] Jodice, P. G. , Adams, E. M. , Lamb, J. , Satgé, Y. & Gleason, J. S. (2019). GoMAMN strategic bird monitoring guidelines: seabirds. In Strategic Bird Monitoring Guidelines for the Northern Gulf of Mexico (Volume 1228), pp. 129–169. Mississippi Agricultural and Forestry Extension Research Bulletin, Starkville, Mississippi.

[brv70143-bib-0095] Jones, A. W. , Dalton, C. M. , Stowe, E. S. & Post, D. M. (2010). Contribution of declining anadromous fishes to the reproductive investment of a common piscivorous seabird, the double‐crested cormorant (*Phalacrocorax auritus*). Auk 127, 696–703.

[brv70143-bib-0096] Jutla, A. S. , Akanda, A. S. , Griffiths, J. K. , Colwell, R. & Islam, S. (2011). Warming oceans, phytoplankton, and river discharge: implications for cholera outbreaks. American Journal of Tropical Medicine and Hygiene 85, 303–310. 21813852 10.4269/ajtmh.2011.11-0181 PMC3144830

[brv70143-bib-0097] Kendall, W. L. , Converse, S. J. , Doherty, P. F. Jr. , Naughton, M. B. , Anders, A. , Hines, J. E. & Flint, E. (2009). Sampling design considerations for demographic studies: a case of colonial seabirds. Ecological Applications 19, 55–68. 19323173 10.1890/07-1072.1

[brv70143-bib-0098] Kowalczyk, N. D. (2015). The foraging and reproductive ecology of a resident, inshore seabird, the little penguin. PhD dissertation, Monash University, Melbourne.

[brv70143-bib-0099] Kowalczyk, N. D. , Reina, R. D. , Preston, T. J. & Chiaradia, A. (2015 *a*). Environmental variability drives shifts in the foraging behaviour and reproductive success of an inshore seabird. Oecologia 178, 967–979. 25894092 10.1007/s00442-015-3294-6

[brv70143-bib-0100] Kowalczyk, N. D. , Reina, R. D. , Preston, T. J. & Chiaradia, A. (2015 *b*). Selective foraging within estuarine plume fronts by an inshore resident seabird. Frontiers in Marine Science 2, 42.

[brv70143-bib-0101] Kralj, J. , Pavlinec, Ž. , Jurinović, L. , Barišić, S. , Martinović, M. , Meštrović, L. , Laušić, M. B. , Ćiković, D. , Tutiš, V. & Lončar, V. (2025). River and sea: foraging range of freshwater and marine common terns. Journal of Ornithology 166, 121–130.

[brv70143-bib-0102] Lamb, J. S. , Satgé, Y. G. & Jodice, P. G. (2020). Seasonal variation in environmental and behavioural drivers of annual‐cycle habitat selection in a nearshore seabird. Diversity and Distributions 26, 254–266.

[brv70143-bib-0103] Lambert, C. , Authier, M. , Doray, M. , Dorémus, G. , Spitz, J. & Ridoux, V. (2018). Decadal stability in top predator habitat preferences in the Bay of Biscay. Progress in Oceanography 166, 109–120.

[brv70143-bib-0104] Lerma, M. , Dehnhard, N. , Castillo‐Guerrero, J. A. & Fernández, G. (2022). Nutritional state variations in a tropical seabird throughout its breeding season. Journal of Comparative Physiology B 192, 775–787. 10.1007/s00360-022-01456-3 PMC9550769 36100755

[brv70143-bib-0105] Lieber, L. , Langrock, R. & Nimmo‐Smith, W. A. M. (2021). A bird's‐eye view on turbulence: seabird foraging associations with evolving surface flow features. Proceedings of the Royal Society B 288, 20210592. 33906396 10.1098/rspb.2021.0592 PMC8079999

[brv70143-bib-0106] Lindenmayer, D. B. , Likens, G. E. , Andersen, A. , Bowman, D. , Bull, C. M. , Burns, E. , Dickman, C. R. , Hoffmann, A. A. , Keith, D. A. & Liddell, M. J. (2012). Value of long‐term ecological studies. Austral Ecology 37, 745–757.

[brv70143-bib-0107] Litzow, M. A. & Piatt, J. F. (2003). Variance in prey abundance influences time budgets of breeding seabirds: evidence from pigeon guillemots *Cepphus columba* . Journal of Avian Biology 34, 54–64.

[brv70143-bib-0108] Lopes, C. S. (2014). The role of vegetation cover and diet in explaining long‐term changes in the breeding population of Little Terns (*Sternula albifrons*) in Ria Formosa, Algarve. Master's thesis, Universidade de Coimbra, Coimbra.

[brv70143-bib-0109] Loredo, S. A. , Orben, R. A. , Suryan, R. M. , Lyons, D. E. , Adams, J. & Stephensen, S. W. (2019). Spatial and temporal diving behavior of non‐breeding common murres during two summers of contrasting ocean conditions. Journal of Experimental Marine Biology and Ecology 517, 13–24.

[brv70143-bib-0110] Louzao, M. , Navarro, J. , Forero, M. G. , Igual, J. M. , Genovart, M. , Hobson, K. A. & Oro, D. (2011). Exploiting the closest productive area: geographical segregation of foraging grounds in a critically endangered seabird. Marine Ecology Progress Series 429, 291–301.

[brv70143-bib-0111] Lukies, K. , Gaskin, C. & Whitehead, E. (2021). The effects of sediment on birds foraging in intertidal and nearshore habitats in Aotearoa New Zealand. Notornis 68, 1–12.

[brv70143-bib-0112] Lyons, D. E. (2010). Bioenergetics‐based predator–prey relationships between piscivorous birds and juvenile salmonids in the Columbia River Estuary. PhD dissertation, Oregon State University, Corvallis.

[brv70143-bib-0113] Lyons, D. E. , Evans, A. F. , Hostetter, N. , Piggott, A. , Weitkamp, L. A. , Good, T. P. , Roby, D. D. , Collis, K. , Loschl, P. J. & Cramer, B. (2014). Factors Influencing Predation on Juvenile Salmonids by Double‐Crested Cormorants in the Columbia River Estuary: A Retrospective Analysis. US Army Corps of Engineers, Portland District.

[brv70143-bib-0114] Maavara, T. , Chen, Q. , Van Meter, K. , Brown, L. E. , Zhang, J. , Ni, J. & Zarfl, C. (2020). River dam impacts on biogeochemical cycling. Nature Reviews Earth & Environment 1, 103–116.

[brv70143-bib-0115] MacDonald, M. A. (2006). The Indirect Effects of Increased Nutrient Inputs on Birds in the UK: A Review. Royal Society for the Protection of Birds, Sandy.

[brv70143-bib-0116] Mallory, M. L. , Robertson, G. J. , Keegan, S. , Pollet, I. L. , Harris, L. N. , Jivan, T. & Provencher, J. F. (2022). Bycatch of loons assessed in coastal Arctic char fisheries in the Canadian Arctic. North American Journal of Fisheries Management 42, 1215–1225.

[brv70143-bib-0117] Malone, T. C. & Newton, A. (2020). The globalization of cultural eutrophication in the coastal ocean: causes and consequences. Frontiers in Marine Science 7, 670.

[brv70143-bib-0118] Markones, N. (2007). Habitat selection of seabirds in a highly dynamic coastal sea: temporal variation and influence of hydrographic features. PhD dissertation, Christian‐Albrechts Universität, Kiel.

[brv70143-bib-0119] Masiá, P. , Ardura, A. & Garcia‐Vazquez, E. (2019). Microplastics in special protected areas for migratory birds in the Bay of Biscay. Marine Pollution Bulletin 146, 993–1001. 31426247 10.1016/j.marpolbul.2019.07.065

[brv70143-bib-0120] Matsumoto, S. , Yamamoto, T. , Kawabe, R. , Ohshimo, S. & Yoda, K. (2016). The Changjiang River discharge affects the distribution of foraging seabirds. Marine Ecology Progress Series 555, 273–277.

[brv70143-bib-0121] Mattern, T. (2020). Modelling Marine Habitat Utilisation by Yellow‐Eyed Penguins along their Mainland Distribution: Baseline Information. Report, Ministry for Primary Industries, Wellington.

[brv70143-bib-0122] Mauco, L. & Favero, M. (2005). The food and feeding biology of common terns wintering in Argentina: influence of environmental conditions. Waterbirds 28, 450–457.

[brv70143-bib-0123] McDuie, F. , Weeks, S. J. & Congdon, B. C. (2018). Oceanographic drivers of near‐colony seabird foraging site use in tropical marine systems. Marine Ecology Progress Series 589, 209–225.

[brv70143-bib-0124] Meijer, L. J. , Van Emmerik, T. , Van Der Ent, R. , Schmidt, C. & Lebreton, L. (2021). More than 1000 rivers account for 80% of global riverine plastic emissions into the ocean. Science Advances 7, eaaz5803. 33931460 10.1126/sciadv.aaz5803 PMC8087412

[brv70143-bib-0125] Milliman, J. D. , Farnsworth, K. , Jones, P. , Xu, K. & Smith, L. (2008). Climatic and anthropogenic factors affecting river discharge to the global ocean, 1951–2000. Global and Planetary Change 62, 187–194.

[brv70143-bib-0126] Mondreti, R. , Davidar, P. , Ryan, P. , Thiebot, J.‐B. & Gremillet, D. (2020). Seabird and cetacean occurrence in the bay of Bengal associated with marine productivity and commercial fishing effort. Marine Ornithology 48, 91–101.

[brv70143-bib-0127] Nagelkerken, I. , Sheaves, M. , Baker, R. & Connolly, R. M. (2015). The seascape nursery: a novel spatial approach to identify and manage nurseries for coastal marine fauna. Fish and Fisheries 16, 362–371.

[brv70143-bib-0128] Naselli‐Flores, L. & Padisák, J. (2023). Ecosystem services provided by marine and freshwater phytoplankton. Hydrobiologia 850, 2691–2706. 35106010 10.1007/s10750-022-04795-y PMC8795964

[brv70143-bib-0129] Newson, S. , Marchant, J. , Sellers, R. , Ekins, G. , Hearn, R. & Burton, N. (2013). Colonisation and range expansion of inland‐breeding cormorants in England. British Birds 106, 737–743.

[brv70143-bib-0130] Northrup, J. M. , Vander Wal, E. , Bonar, M. , Fieberg, J. , Laforge, M. P. , Leclerc, M. , Prokopenko, C. M. & Gerber, B. D. (2022). Conceptual and methodological advances in habitat‐selection modeling: guidelines for ecology and evolution. Ecological Applications 32, e02470. 34626518 10.1002/eap.2470 PMC9285351

[brv70143-bib-0131] Nunes, G. T. , Efe, M. A. , Barreto, C. T. , Gaiotto, J. V. , Silva, A. B. , Vilela, F. , Roy, A. , Bertrand, S. , Costa, P. G. & Bianchini, A. (2022). Ecological trap for seabirds due to the contamination caused by the Fundão dam collapse, Brazil. Science of the Total Environment 807, 151486. 34742806 10.1016/j.scitotenv.2021.151486

[brv70143-bib-0132] O'Keeffe, J. , Bukaciński, D. , Bukacińska, M. , Piniewski, M. & Okruszko, T. (2023). Future of birds nesting on river islands in the conditions of hydrological variability caused by climate change. Ecohydrology & Hydrobiology 23, 1–10.

[brv70143-bib-0133] O'Leary, J. K. , Micheli, F. , Airoldi, L. , Boch, C. , De Leo, G. , Elahi, R. , Ferretti, F. , Graham, N. A. , Litvin, S. Y. & Low, N. H. (2017). The resilience of marine ecosystems to climatic disturbances. Bioscience 67, 208–220.

[brv70143-bib-0134] Orgeret, F. , Thiebault, A. , Kovacs, K. M. , Lydersen, C. , Hindell, M. A. , Thompson, S. A. , Sydeman, W. J. & Pistorius, P. A. (2022). Climate change impacts on seabirds and marine mammals: the importance of study duration, thermal tolerance and generation time. Ecology Letters 25, 218–239. 34761516 10.1111/ele.13920

[brv70143-bib-0135] Page, M. J. , Mckenzie, J. E. , Bossuyt, P. M. , Boutron, I. , Hoffmann, T. C. , Mulrow, C. D. , Shamseer, L. , Tetzlaff, J. M. , Akl, E. A. & Brennan, S. E. (2021). The PRISMA 2020 statement: an updated guideline for reporting systematic reviews. British Medical Journal 372, n71. 33782057 10.1136/bmj.n71 PMC8005924

[brv70143-bib-0136] Parrish, R. H. , Nelson, C. S. & Bakun, A. (1981). Transport mechanisms and reproductive success of fishes in the California current. Biological Oceanography 1, 175–203.

[brv70143-bib-0137] Pastran, S. A. , Drever, M. C. & Lank, D. B. (2021). Marbled murrelets prefer stratified waters close to freshwater inputs in Haida Gwaii, British Columbia, Canada. Ornithological Applications 123, duab043.

[brv70143-bib-0138] Peck‐Richardson, A. G. , Lyons, D. E. , Roby, D. D. , Cushing, D. A. & Lerczak, J. A. (2018). Three‐dimensional foraging habitat use and niche partitioning in two sympatric seabird species, *Phalacrocorax auritus* and *P. Penicillatus* . Marine Ecology Progress Series 586, 251–264.

[brv70143-bib-0139] Phillips, E. M. , Horne, J. K. & Zamon, J. E. (2017). Predator–prey interactions influenced by a dynamic river plume. Canadian Journal of Fisheries and Aquatic Sciences 74, 1375–1390.

[brv70143-bib-0140] Phillips, E. M. , Horne, J. K. & Zamon, J. E. (2021). Characterizing juvenile salmon predation risk during early marine residence. PLoS One 16, e0247241. 33606791 10.1371/journal.pone.0247241 PMC7894896

[brv70143-bib-0141] Phillips, E. M. , Horne, J. K. , Adams, J. & Zamon, J. E. (2018). Selective occupancy of a persistent yet variable coastal river plume by two seabird species. Marine Ecology Progress Series 594, 245–261.

[brv70143-bib-0142] Phillips, J. A. , Guilford, T. & Fayet, A. L. (2023). How do resource distribution and taxonomy affect the use of dual foraging in seabirds? A review. Behavioral Ecology 34, 769–779. 37744167 10.1093/beheco/arad052 PMC10516677

[brv70143-bib-0143] Piatt, J. F. , Harding, A. M. , Shultz, M. , Speckman, S. G. , Van Pelt, T. I. , Drew, G. S. & Kettle, A. B. (2007). Seabirds as indicators of marine food supplies: Cairns revisited. Marine Ecology Progress Series 352, 221–234.

[brv70143-bib-0144] Ponton, D. E. , Lavoie, R. A. , Leclerc, M. , Bilodeau, F. , Planas, D. & Amyot, M. (2021). Understanding food web mercury accumulation through trophic transfer and carbon processing along a river affected by recent run‐of‐river dams. Environmental Science & Technology 55, 2949–2959. 33534545 10.1021/acs.est.0c07015

[brv70143-bib-0145] Poupart, T. A. , Waugh, S. M. , Bost, C. , Bost, C.‐A. , Dennis, T. , Lane, R. , Rogers, K. , Sugishita, J. , Taylor, G. A. & Wilson, K.‐J. (2017). Variability in the foraging range of *Eudyptula minor* across breeding sites in central New Zealand. New Zealand Journal of Zoology 44, 225–244.

[brv70143-bib-0146] Power, A. , Newton, S. , Burke, B. , Tierney, D. & O'Connor, I. (2023). Seabirds. Marine Institute, Galway.

[brv70143-bib-0147] Precheur, C. , Barbraud, C. , Martail, F. , Mian, M. , Nicolas, J. C. , Brithmer, R. , Belfan, D. , Conde, B. & Bretagnolle, V. (2016). Some like it hot: effect of environment on population dynamics of a small tropical seabird in the Caribbean region. Ecosphere 7, e01461.

[brv70143-bib-0148] Price, C. A. , Hartmann, K. , Emery, T. J. , Woehler, E. J. , Mcmahon, C. R. & Hindell, M. A. (2020). Climate variability and breeding parameters of a transhemispheric migratory seabird over seven decades. Marine Ecology Progress Series 642, 191–205.

[brv70143-bib-0149] Provencher, J. F. , Bond, A. L. , Avery‐Gomm, S. , Borrelle, S. B. , Rebolledo, E. L. B. , Hammer, S. , Kühn, S. , Lavers, J. L. , Mallory, M. L. & Trevail, A. (2017). Quantifying ingested debris in marine megafauna: a review and recommendations for standardization. Analytical Methods 9, 1454–1469.

[brv70143-bib-0150] Ramírez, F. , Afán, I. , Davis, L. S. & Chiaradia, A. (2017). Climate impacts on global hot spots of marine biodiversity. Science Advances 3, e1601198. 28261659 10.1126/sciadv.1601198 PMC5321448

[brv70143-bib-0151] Ramos, J. A. & Pereira, L. (2022). Seabird Biodiversity and Human Activities, Edition (Volume 1). CRC Press, Boca Raton.

[brv70143-bib-0152] Raoult, V. , Phillips, A. A. , Nelson, J. , Niella, Y. , Skinner, C. , Tilcock, M. B. , Burke, P. J. , Szpak, P. , James, W. R. & Harrod, C. (2024). Why aquatic scientists should use sulfur stable isotope ratios (ẟ34S) more often. Chemosphere 355, 141816. 38556184 10.1016/j.chemosphere.2024.141816

[brv70143-bib-0153] Raphael, M. G. , Shirk, A. J. , Falxa, G. A. & Pearson, S. F. (2015). Habitat associations of marbled murrelets during the nesting season in nearshore waters along the Washington to California coast. Journal of Marine Systems 146, 17–25.

[brv70143-bib-0154] Rattner, B. A. , Wazniak, C. E. , Lankton, J. S. , Mcgowan, P. C. , Drovetski, S. V. & Egerton, T. A. (2022). Review of harmful algal bloom effects on birds with implications for avian wildlife in the Chesapeake Bay region. Harmful Algae 120, 102319. 36470599 10.1016/j.hal.2022.102319

[brv70143-bib-0155] Rebstock, G. A. & Boersma, P. D. (2018). Oceanographic conditions in wintering grounds affect arrival date and body condition in breeding female Magellanic penguins. Marine Ecology Progress Series 601, 253–267.

[brv70143-bib-0156] Ribic, C. A. , Davis, R. , Hess, N. & Peake, D. (1997). Distribution of seabirds in the northern Gulf of Mexico in relation to mesoscale features: initial observations. ICES Journal of Marine Science 54, 545–551.

[brv70143-bib-0157] Robuck, A. R. , Cantwell, M. G. , Mccord, J. P. , Addison, L. M. , Pfohl, M. , Strynar, M. J. , Mckinney, R. , Katz, D. R. , Wiley, D. N. & Lohmann, R. (2020). Legacy and novel per‐ and polyfluoroalkyl substances in juvenile seabirds from the US Atlantic Coast. Environmental Science & Technology 54, 12938–12948. 32894676 10.1021/acs.est.0c01951 PMC7700771

[brv70143-bib-0158] Rodríguez, A. , Arcos, J. M. , Bretagnolle, V. , Dias, M. P. , Holmes, N. D. , Louzao, M. , Provencher, J. , Raine, A. F. , Ramirez, F. & Rodriguez, B. (2019). Future directions in conservation research on petrels and shearwaters. Frontiers in Marine Science 6, 94.

[brv70143-bib-0159] Ronconi, R. & Burger, A. (2011). Foraging space as a limited resource: inter‐ and intra‐specific competition among sympatric pursuit‐diving seabirds. Canadian Journal of Zoology 89, 356–368.

[brv70143-bib-0160] Ropert‐Coudert, Y. , Chiaradia, A. , Ainley, D. , Barbosa, A. , Boersma, P. D. , Brasso, R. , Dewar, M. , Ellenberg, U. , García‐Borboroglu, P. & Emmerson, L. (2019). Happy feet in a hostile world? The future of penguins depends on proactive management of current and expected threats. Frontiers in Marine Science 6, 248.

[brv70143-bib-0161] Ropert‐Coudert, Y. , Kato, A. & Chiaradia, A. (2009). Impact of small‐scale environmental perturbations on local marine food resources: a case study of a predator, the little penguin. Proceedings of the Royal Society B: Biological Sciences 276, 4105–4109. 10.1098/rspb.2009.1399 PMC2821355 19729454

[brv70143-bib-0162] Royan, A. (2015). The influence of river flow on the distribution and community organisation of river birds. PhD dissertation, University of Birmingham, Birmingham.

[brv70143-bib-0163] Royan, A. , Hannah, D. M. , Reynolds, S. J. , Noble, D. G. & Sadler, J. P. (2014). River birds' response to hydrological extremes: new vulnerability index and conservation implications. Biological Conservation 177, 64–73.

[brv70143-bib-0164] Russell, I. C. , Cook, A. C. , Ives, M. J. & Davison, P. I. (2022). The diet of two sympatric great cormorant *Phalacrocorax carbo* subspecies wintering at freshwater fishery sites in England and Wales. Ardea 109, 443–456.

[brv70143-bib-0165] Ryan, P. G. (2015). A brief history of marine litter research. In Marine Anthropogenic Litter, pp. 1–25. Springer International Publishing, Cham.

[brv70143-bib-0166] Santora, J. A. , Schroeder, I. D. , Field, J. C. , Wells, B. K. & Sydeman, W. J. (2014). Spatio‐temporal dynamics of ocean conditions and forage taxa reveal regional structuring of seabird–prey relationships. Ecological Applications 24, 1730–1747. 29210234 10.1890/13-1605.1

[brv70143-bib-0167] Scales, K. L. , Miller, P. I. , Hawkes, L. A. , Ingram, S. N. , Sims, D. W. & Votier, S. C. (2014). On the front line: frontal zones as priority at‐sea conservation areas for mobile marine vertebrates. Journal of Applied Ecology 51, 1575–1583.

[brv70143-bib-0168] Schwemmer, P. , Adler, S. , Guse, N. , Markones, N. & Garthe, S. (2009). Influence of water flow velocity, water depth and colony distance on distribution and foraging patterns of terns in the Wadden Sea. Fisheries Oceanography 18, 161–172.

[brv70143-bib-0169] Scott, B. E. , Sharples, J. , Wanless, S. , Ross, O. , Frederiksen, M. & Daunt, F. (2006). The use of biologically meaningful oceanographic indices to separate the effects of climate and fisheries on seabird breeding success. In Conservation Biology Series (Volume 12), p. 46. Cambridge.

[brv70143-bib-0170] Sebastiano, M. , Costantini, D. , Eens, M. , Pineau, K. , Bustamante, P. & Chastel, O. (2022). Possible interaction between exposure to environmental contaminants and nutritional stress in promoting disease occurrence in seabirds from French Guiana: a review. Regional Environmental Change 22, 63.

[brv70143-bib-0171] Seher, V. L. , Holzman, B. A. , Hines, E. , Bradley, R. W. , Warzybok, P. & Becker, B. H. (2022). Ocean‐influenced estuarine habitat buffers high interannual variation in seabird reproductive success. Marine Ecology Progress Series 689, 155–167.

[brv70143-bib-0172] Shealer, D. A. (2002). Foraging behavior and food of seabirds. Biology of Marine Birds 14, 137–177.

[brv70143-bib-0173] Sherman, K. & Hamukuaya, H. (2016). Sustainable development of the world's large marine ecosystems. In Environmental Development (Volume 17), pp. 1–6. Elsevier, Amsterdam.

[brv70143-bib-0174] Signa, G. , Mazzola, A. & Vizzini, S. (2021). Seabird influence on ecological processes in coastal marine ecosystems: an overlooked role? A critical review. Estuarine, Coastal and Shelf Science 250, 107164.

[brv70143-bib-0175] Skov, H. & Prins, E. (2001). Impact of estuarine fronts on the dispersal of piscivorous birds in the German bight. Marine Ecology Progress Series 214, 279–287.

[brv70143-bib-0176] Soanes, L. , Green, J. , Bolton, M. , Milligan, G. , Mukhida, F. & Halsey, L. (2021). Linking foraging and breeding strategies in tropical seabirds. Journal of Avian Biology 52, e02670.

[brv70143-bib-0177] Sulc, A. (2019). Chlorophyll‐a patterns during Magellanic penguin breeding at Punta Tombo. Academic thesis, University of Washington, Seattle.

[brv70143-bib-0178] Suzuki, Y. (2012). Piscivorous colonial waterbirds in the Columbia River estuary: demography, dietary contaminants, and management. PhD dissertation, Oregon State University, Corvallis.

[brv70143-bib-0179] Suzuki, Y. , Bishop, M. A. , Roby, D. D. & Bixler, K. S. (2019). Colony connectivity and the rapid growth of a Caspian tern (*Hydroprogne caspia*) colony on Alaska's Copper River Delta, USA. Waterbirds 42, 1–7.

[brv70143-bib-0180] Tavares, D. C. , Fulgencio De Moura, J. & Siciliano, S. (2016). Environmental predictors of seabird wrecks in a tropical coastal area. PLoS One 11, e0168717. 27992578 10.1371/journal.pone.0168717 PMC5161483

[brv70143-bib-0181] Teng, J. , Jakeman, A. J. , Vaze, J. , Croke, B. F. , Dutta, D. & Kim, S. (2017). Flood inundation modelling: a review of methods, recent advances and uncertainty analysis. Environmental Modelling & Software 90, 201–216.

[brv70143-bib-0182] The Ad Hoc Group , Vörösmarty, C. , Askew, A. , Grabs, W. , Barry, R. G. , Birkett, C. , Döll, P. , Goodison, B. , Hall, A. , Jenne, R. , Kitaev, L. , Landwehr, J. , Keeler, M. , Leavesley, G. , Schaake, J. , *et al*. (2001). Global water data: a newly endangered species. In Eos, Transactions American Geophysical Union (Volume 82), pp. 54–58. American Geophysical Union, Washington, DC.

[brv70143-bib-0183] Thibault, M. , Houlbrèque, F. , Lorrain, A. & Vidal, E. (2019). Seabirds: sentinels beyond the oceans. Science 366, 813. 10.1126/science.aaz7665 31727822

[brv70143-bib-0184] Thibault, M. , Weiss, L. , Fernandez, R. , Avargues, N. , Jaquemet, S. , Lebreton, L. , Garnier, J. , Jaeger, A. , Royer, S.‐J. & Cartraud, A. (2024). Barau's petrel, *Pterodroma baraui*, as a bioindicator of plastic pollution in the south‐West Indian Ocean: a multifaceted approach. Marine Environmental Research 202, 106709. 39260181 10.1016/j.marenvres.2024.106709

[brv70143-bib-0185] Thorne, L. H. , Fuirst, M. , Veit, R. & Baumann, Z. (2021). Mercury concentrations provide an indicator of marine foraging in coastal birds. Ecological Indicators 121, 106922.

[brv70143-bib-0186] Ummenhofer, C. C. & Meehl, G. A. (2017). Extreme weather and climate events with ecological relevance: a review. Philosophical Transactions of the Royal Society B: Biological Sciences 372, 20160135. 10.1098/rstb.2016.0135 PMC5434087 28483866

[brv70143-bib-0187] Van Sebille, E. , Aliani, S. , Law, K. L. , Maximenko, N. , Alsina, J. M. , Bagaev, A. , Bergmann, M. , Chapron, B. , Chubarenko, I. & Cózar, A. (2020). The physical oceanography of the transport of floating marine debris. Environmental Research Letters 15, 023003.

[brv70143-bib-0188] Vanstreels, R. E. , Uhart, M. M. & Work, T. M. (2023). Health and diseases. In Conservation of Marine Birds. Elsevier, Amsterdam.

[brv70143-bib-0189] Waggitt, J. J. , Cazenave, P. W. , Howarth, L. M. , Evans, P. G. , Van Der Kooij, J. & Hiddink, J. G. (2018). Combined measurements of prey availability explain habitat selection in foraging seabirds. Biology Letters 14, 20180348. 30068542 10.1098/rsbl.2018.0348 PMC6127128

[brv70143-bib-0190] Waggitt, J. , Torres, R. & Fraser, S. (2020). Foraging seabirds respond to an intermittent meteorological event in a coastal environment. Marine Ornithology 48, 123–131.

[brv70143-bib-0191] Webb, T. J. (2012). Marine and terrestrial ecology: unifying concepts, revealing differences. Trends in Ecology & Evolution 27, 535–541. 22795608 10.1016/j.tree.2012.06.002

[brv70143-bib-0192] Weimerskirch, H. (2007). Are seabirds foraging for unpredictable resources? Deep Sea Research Part II: Topical Studies in Oceanography 54, 211–223.

[brv70143-bib-0193] Wells, B. K. , Santora, J. A. , Henderson, M. J. , Warzybok, P. , Jahncke, J. , Bradley, R. W. , Huff, D. D. , Schroeder, I. D. , Nelson, P. & Field, J. C. (2017). Environmental conditions and prey‐switching by a seabird predator impact juvenile salmon survival. Journal of Marine Systems 174, 54–63.

[brv70143-bib-0194] Wells, M. R. , Coggan, T. L. , Stevenson, G. , Singh, N. , Askeland, M. , Lea, M.‐A. , Philips, A. & Carver, S. (2023). Per‐ and polyfluoroalkyl substances (PFAS) in little penguins and associations with urbanisation and health parameters. Science of the Total Environment 912, 169084. 38056658 10.1016/j.scitotenv.2023.169084

[brv70143-bib-0195] Whittington, P. A. , Crawford, R. J. , Martin, A. P. , Randall, R. M. , Brown, M. , Ryan, P. G. , Dyer, B. M. , Harrison, K. H. , Huisamen, J. & Makhado, A. B. (2016). Recent trends of the kelp Gull (*Larus dominicanus*) in South Africa. Waterbirds 39, 99–113.

[brv70143-bib-0196] Wolf, S. G. , Snyder, M. A. , Sydeman, W. J. , Doak, D. F. & Croll, D. A. (2010). Predicting population consequences of ocean climate change for an ecosystem sentinel, the seabird Cassin's auklet. Global Change Biology 16, 1923–1935.

[brv70143-bib-0197] Wu, Q. , Ke, L. , Wang, J. , Pavelsky, T. M. , Allen, G. H. , Sheng, Y. , Duan, X. , Zhu, Y. , Wu, J. & Wang, L. (2023). Satellites reveal hotspots of global river extent change. Nature Communications 14, 1587. 10.1038/s41467-023-37061-3 PMC10033638 36949069

[brv70143-bib-0198] Yen, P. P. , Huettmann, F. & Cooke, F. (2004). A large‐scale model for the at‐sea distribution and abundance of marbled murrelets (*Brachyramphus marmoratus*) during the breeding season in coastal British Columbia, Canada. Ecological Modelling 171, 395–413.

[brv70143-bib-0199] Zamon, J. E. , Phillips, E. M. & Guy, T. J. (2014). Marine bird aggregations associated with the tidally‐driven plume and plume fronts of the Columbia River. Deep Sea Research Part II: Topical Studies in Oceanography 107, 85–95.

[brv70143-bib-0200] Zeiringer, B. , Seliger, C. , Greimel, F. & Schmutz, S. (2018). River hydrology, flow alteration, and environmental flow. In Riverine Ecosystem Management: Science for Governing Towards a Sustainable Future, pp. 67–89. Springer International Publishing, Cham.

